# Noncanonical CDK4 signaling rescues diabetes in a mouse model by promoting **β** cell differentiation

**DOI:** 10.1172/JCI166490

**Published:** 2023-09-15

**Authors:** Rachel E. Stamateris, Huguet V. Landa-Galvan, Rohit B. Sharma, Christine Darko, David Redmond, Sushil G. Rane, Laura C. Alonso

**Affiliations:** 1 MD/PhD Program, University of Massachusetts Medical School, Worcester, Massachusetts, USA.; 2 Division of Endocrinology, Diabetes and Metabolism and the Joan and Sanford I. Weill Center for Metabolic Health and; 3 Hartman Institute for Therapeutic Regenerative Medicine, Division of Regenerative Medicine, Department of Medicine, Weill Cornell Medicine, New York, New York, USA.; 4 Integrative Cellular Metabolism Section, Diabetes, Endocrinology and Obesity Branch, National Institute for Diabetes, Digestive and Kidney Diseases, NIH, Bethesda, Maryland, USA.

**Keywords:** Endocrinology, Metabolism, Beta cells, Cell cycle, Insulin

## Abstract

Expanding β cell mass is a critical goal in the fight against diabetes. CDK4, an extensively characterized cell cycle activator, is required to establish and maintain β cell number. β cell failure in the IRS2-deletion mouse type 2 diabetes model is, in part, due to loss of CDK4 regulator cyclin D2. We set out to determine whether replacement of endogenous CDK4 with the inhibitor-resistant mutant CDK4-R24C rescued the loss of β cell mass in IRS2-deficient mice. Surprisingly, not only β cell mass but also β cell dedifferentiation was effectively rescued, despite no improvement in whole body insulin sensitivity. Ex vivo studies in primary islet cells revealed a mechanism in which CDK4 intervened downstream in the insulin signaling pathway to prevent FOXO1-mediated transcriptional repression of critical β cell transcription factor *Pdx1*. FOXO1 inhibition was not related to E2F1 activity, to FOXO1 phosphorylation, or even to FOXO1 subcellular localization, but rather was related to deacetylation and reduced FOXO1 abundance. Taken together, these results demonstrate a differentiation-promoting activity of the classical cell cycle activator CDK4 and support the concept that β cell mass can be expanded without compromising function.

## Graphical abstract

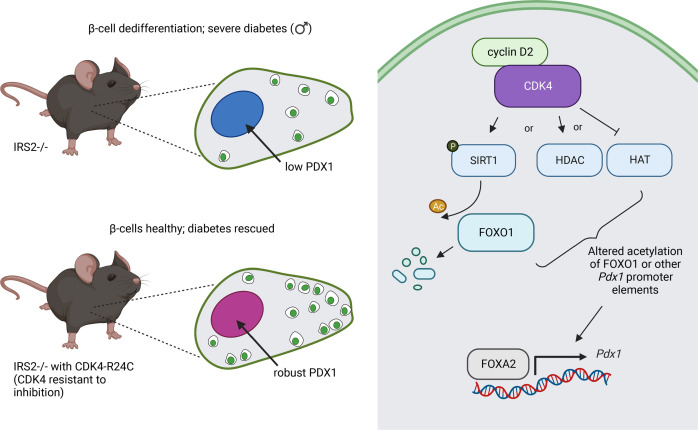

## Introduction

An increase in endogenous pancreatic β cell mass and function would address the harms caused by diabetes in people with insulin deficiency. Nutritional exposures such as hyperglycemia (1), high-fat diet (2, 3), and other nutrient excess paradigms (4) promote β cell proliferation via mitogenic inputs that converge on downstream signaling to traverse the G1/S transition of the cell cycle (3, 5, 6). On the other hand, insulin secretory capacity can be lost through β cell death (7) and dedifferentiation (8, 9); some data suggest the reduction of β cell mass in human type 2 diabetes (T2D) may have been overestimated (10). Nonetheless, a critical barrier in the β cell regeneration field is that strategies that increase replication may also lead to dedifferentiation (8, 9, 11).

Whole-body deletion of insulin receptor substrate–2 (IRS-2) in mice causes a T2D-like syndrome due to reduced β cell function and mass in the face of marked insulin resistance (12, 13). Distal insulin signaling pathway member forkhead box protein O1 (FOXO1) causes dedifferentiation in this model via suppression of key β cell factor *Pdx1* (9, 14). *Irs2^–/–^* β cells also have an impaired proliferative response to glucose due to reduced induction of cyclin D2, and restoring cyclin D2 abundance rescues proliferation to normal levels (15). Cyclin D2, a driver of postnatal β cell expansion and β cell compensation for insulin resistance (1, 16–20), binds to and activates cyclin-dependent kinase (CDK) family members CDK4 and 6 to hyperphosphorylate the retinoblastoma (Rb) protein and derepress E2F transcription factors. CDK4 is critical for murine β cells; deletion of CDK4 leads to a dramatic reduction in β cell mass (21, 22). We postulated that if loss of cyclin D2/CDK4 activity in β cells is a primary cause of diabetes in IRS2-null mice, then activating CDK4 in vivo (22) might counteract the diabetogenic phenotype in these mice. Specifically, we hypothesized that the *Cdk4-R24C* nucleotide substitution, which renders CDK4 uninhibitable by INK-family cell cycle inhibitors (23, 24), would rescue lost β cell proliferation and mass in mice lacking IRS2.

Here, we report that replacing both alleles of *Cdk4* with *Cdk4-R24C* rescued glucose intolerance in IRS2-null mice without improving insulin sensitivity. β cell mass and proliferation defects were rescued, as predicted. Surprisingly, *Cdk4-R24C* also corrected β cell dedifferentiation, with full restoration of PDX1 and FOXO1 localization and cellular morphology. Intriguingly, CDK4 relieved FOXO1-mediated *Pdx1* repression, but the effect did not correlate with subcellular localization of FOXO1, nor did it require phosphorylation of FOXO1. Rather, the data were consistent with a model in which CDK4/cyclin D2 modulated histone deacetylase (HDAC) or histone acetyltransferase (HAT) activity to suppress negative effects of FOXO1 in the setting of IRS2 deficiency. Taken together, this work highlights a role for CDK4 in the β cell outside of cell cycle regulation, supporting the mature β cell phenotype by derepressing *Pdx1* expression via the insulin signaling mediator FOXO1.

## Results

### Homozygous replacement of WT Cdk4 with Cdk4-R24C rescued diabetes in Irs2^–/–^ male mice.

**Figure 1:**
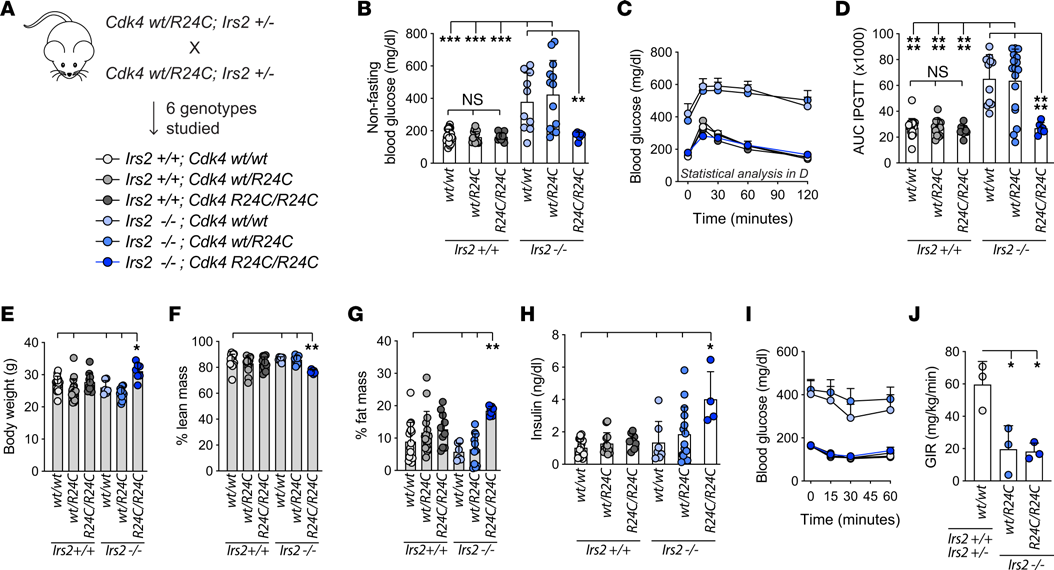
Homozygous replacement of *Cdk4* with *Cdk4-R24C* rescued diabetes in *Irs2^–/–^* male mice. (**A**) Breeding dams and sires doubly heterozygous for *Cdk4-R24C* (whole body knockin) and the *Irs2^–/–^* allele (whole-body KO) produced littermate 8–12 week experimental mice of the genotypes shown. *Irs2^+/–^* progeny were not studied. (**B**–**D**) Nonfasting blood glucose (*n* ≥ 7) (**B**), and blood glucose time course (*n* ≥ 6) (**C**) and AUC (*n* ≥ 6) (**D**) after i.p. glucose challenge. (**E**–**G**) Body composition analysis by 1H-MRS Echo-MRI with body weight (*n* ≥ 6) (**E**), percentage lean mass (*n* ≥ 6) (**F**), and percentage fat mass (*n* ≥ 6) (**G**). Circulating plasma insulin levels (*n* ≥ 4) (**H**) were measured by ELISA. Glycemic response to i.p. insulin challenge (*n* ≥ 6) (**I**) was difficult to interpret due to markedly different baseline values. (**J**) Hyperinsulinemic euglycemic clamp showed that insulin resistance in *Irs2^–/–^* mice was not rescued by homozygous *Cdk4-R24C* (*n* = 3). Only male mice shown here; for females see Supplemental Figure 1. Statistics by 1-way (ANOVA with Tukey’s posthoc test. *P < 0.05; **P < 0.01; ***P < 0.001; ****P < 0.0001.

To test whether activating CDK4 rescued lost β cell mass in IRS2-null mice, we mated mice doubly heterozygous in all tissues (Figure 1A) for a loss-of-function allele of *Irs2* (12) and a gain-of-function allele of *Cdk4*, *Cdk4-R24C*. *Cdk4-R24C* contains an engineered point mutation at the endogenous *Cdk4* locus rendering CDK4 resistant to inhibition by INK-family inhibitors (22). Progeny of matings in which both dam and sire were *Irs2^+/–^; Cdk4-WT/R24C* included the 6 genotypes of interest: *Irs2* WT (*^+/+^*) or KO (*^–/–^*) that were WT (*WT/WT*), heterozygous (*WT/R24C*), or homozygous (*R24C/R24C*) for *Cdk4-R24C* at the *Cdk4* locus. We did not study mice heterozygous for *Irs2* deletion, given their normal glucose homeostasis in our colony (15). Both males and females were analyzed.

As expected (12, 13), male *Irs2^–/–^; Cdk4-WT/WT* mice had elevated nonfasting blood glucose (Figure 1B) and were markedly glucose intolerant (Figure 1, C and D). Female *Irs2^–/–^* mice, as previously reported, had only a slight, nonsignificant elevation in fasting blood glucose and glucose tolerance even when studied at a slightly older age or after 4 weeks of high-fat feeding (Supplemental Figure 1, A–G; supplemental material available online with this article; https://doi.org/10.1172/JCI166490DS1). Remarkably, homozygous replacement of *Cdk4-WT* with *Cdk4-R24C* in *Irs2*^–/–^ males completely restored random nonfasting and postchallenge glucose curves to levels equivalent to nondiabetic controls (Figure 1, B–D). *Cdk4* heterozygous (*WT/R24C*) replacement did not lead to discernible improvement in glucose tolerance in *Irs2*^–/–^ mice (Figure 1, B–D). Thus, whole-body homozygous, but not heterozygous, replacement of *Cdk4-WT* with *Cdk4-R24C* provided effective protection against diabetes in male mice lacking IRS2.

### Cdk4-R24C rescue of Irs2^–/–^ diabetes was not due to improved insulin sensitivity.

In addition to islet growth benefits (21, 22, 25), CDK4 was reported to enhance insulin sensitivity in insulin responsive tissues such as adipose and liver (26, 27). We hypothesized that restoration of glucose metabolism in *Irs2^–/–^* male mice was due to improved insulin sensitivity, increased insulin secretory capacity, or both. *Irs2^–/–^;CdK4-R24C/R24C* males were heavier than littermate controls, with increased percentage fat mass and reduced lean mass (Figure 1, E–G). This effect was not observed in high fat–fed females (Supplemental Figure 1, H–J). Plasma insulin measurements confirmed hyperinsulinemia in *Irs2^–/–^;Cdk4-R24C/R24C* males (Figure 1H), suggesting rescue of insulin secretory capacity in the context of at least some residual insulin resistance. Insulin tolerance tests were difficult to interpret due to different baseline glucose levels (Figure 1I); expressing the data as percentage baseline glucose did not help clarify whether insulin resistance was rescued (data not shown). Hyperinsulinemic euglycemic clamp revealed that the glucose infusion rate, low in *Irs2^–/–^* mice, was not rescued by homozygous *R24C* alleles, suggesting that impaired insulin sensitivity in IRS2-null mice was not improved by *Cdk4-R24C* (Figure 1J). The increased fat mass in *Irs2^–/–^;Cdk4-R24C/R24C* mice (Figure 1G) could be consistent with enhanced insulin-responsive lipogenesis in adipocytes, but, if present, this was insufficient to rescue whole-body insulin resistance. Taken together, these data suggest that the rescue of diabetes in IRS2-null mice by *Cdk4-R24C* was due to correction of insulin deficiency rather than insulin responsiveness.

### Cdk4-R24C restored β cell mass and increased β cell proliferation in Irs2^–/–^ male mice.

**Figure 2:**
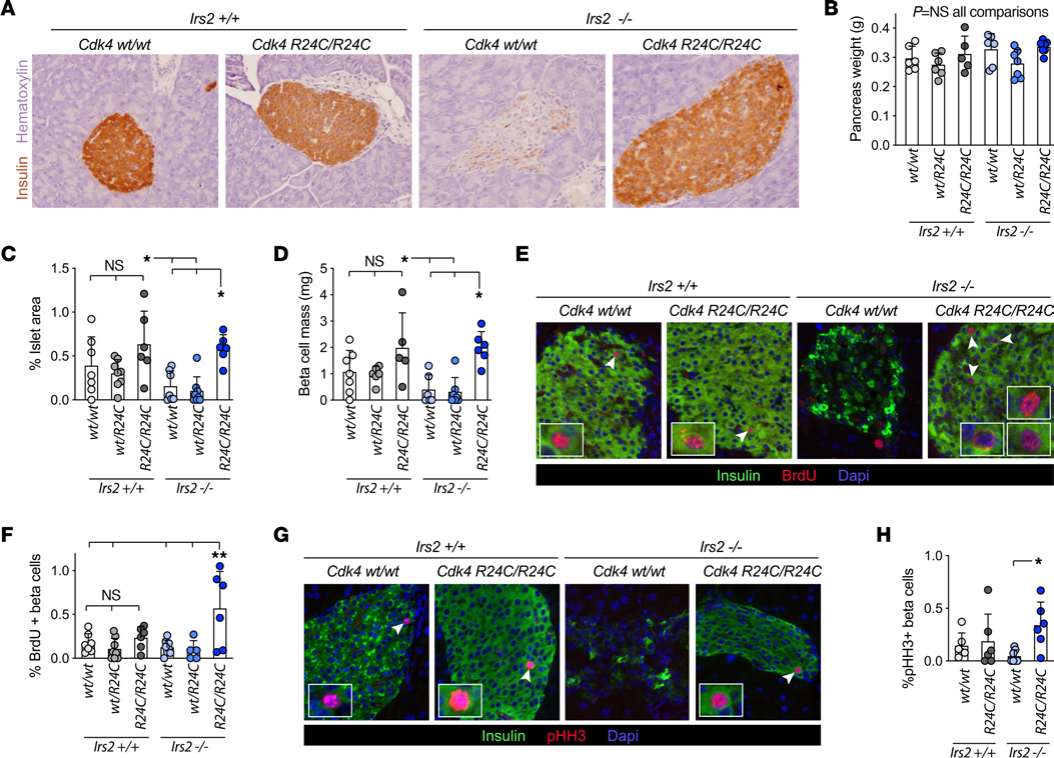
*Cdk4-R24C* rescued β cell mass and proliferation in *Irs2^–/–^* mice. (**A**) Pancreas of adult male mice immunostained for insulin (brown) and counterstained with hematoxylin (purple). (**B**–**D**) (*n* ≥ 5): Wet weight of dissected pancreas before fixation (**B**) was multiplied by the percentage islet area (**C**) to estimate β cell mass (**D**). (**E** and **F**) (*n* ≥ 5) Images from BrdU (red), insulin (green) and Dapi (blue) stained sections (**E**) were used to quantify the percentage of insulin-positive cells that had BrdU^+^ nuclei (**F**). (**G** and **H**) (*n* ≥ 6) Images from pHH3 (red), insulin (green) and Dapi (blue) stained sections (**G**) were used to quantify the percentage of insulin^+^ cells that had pHH3^+^ nuclei (**H**). Images in **A**, **E**, and **G** are representative of *n* ≥ 5 experiments. Original magnification, ×200 (**A**, **E**, and **G**). Statistics by 1-way ANOVA with Tukey’s posthoc test. *P < 0.05; **P < 0.01; ***P < 0.001; ****P < 0.0001.

Since homozygous *Cdk4-R24C* alleles rescued glucose intolerance in male mice by correcting insulin deficiency, we predicted that β cell function, mass, or both were restored in *Irs2^–/–^;Cdk4-R24C/R24C* mice. As expected, pancreas sections showed that islets in male *Irs2^–/–^;Cdk4-WT/WT* mice had less immunoreactivity for insulin than controls. In contrast, male *Irs2^–/–^;Cdk4-R24C/R24C* mice had fully rescued islet morphology and insulin staining (Figure 2A). Pancreas weight was similar among genotypes (Figure 2B), but β cell mass estimated using a ratiometric approach showed a complete rescue of percentage insulin-stained area and β cell mass in *Cdk4-R24C* homozygous, but not heterozygous, male mice (Figure 2, C and D). Since CDK4 is a positive regulator of the cell cycle, we hypothesized that the expanded β cell mass in *Cdk4*-*R24C* homozygous mice was due to increased β cell proliferation. In pancreas sections from male mice, the percentage β cells with nuclear BrdU (Figure 2, E and F) or pHH3 (Figure 2, G and H) labeling was increased in *Cdk4-R24C* homozygous mice over diabetic controls. BrdU incorporation was only significantly increased by *Cdk4-R24C* in the *Irs2^–/–^* context, suggesting the proliferation was amplified by insulin resistance. Analysis of pancreas sections also showed a nonsignificant reduction in β cell apoptosis in *Irs2^–/–^;Cdk4-R24C/R24C* male mice compared with controls (Supplemental Figure 2). Thus, expanded β cell mass due to increased proliferation, and possibly reduced cell death, likely contributed to the diabetes rescue.

We considered the possibility that the *Cdk4-R24C* variant might have altered RNA or protein kinetics that resulted in overexpression of the *Cdk4-R24C* variant relative to the WT *Cdk4* isoform. To test this possibility we expressed these variants in mouse islet cells using equal multiplicity of infection (MOI) of adenovirus. Both variants produced similar amounts of protein by immunoblot (Supplemental Figure 3).

### Cdk4-R24C restored islet morphological defects in IRS2-null male mice.

**Figure 3:**
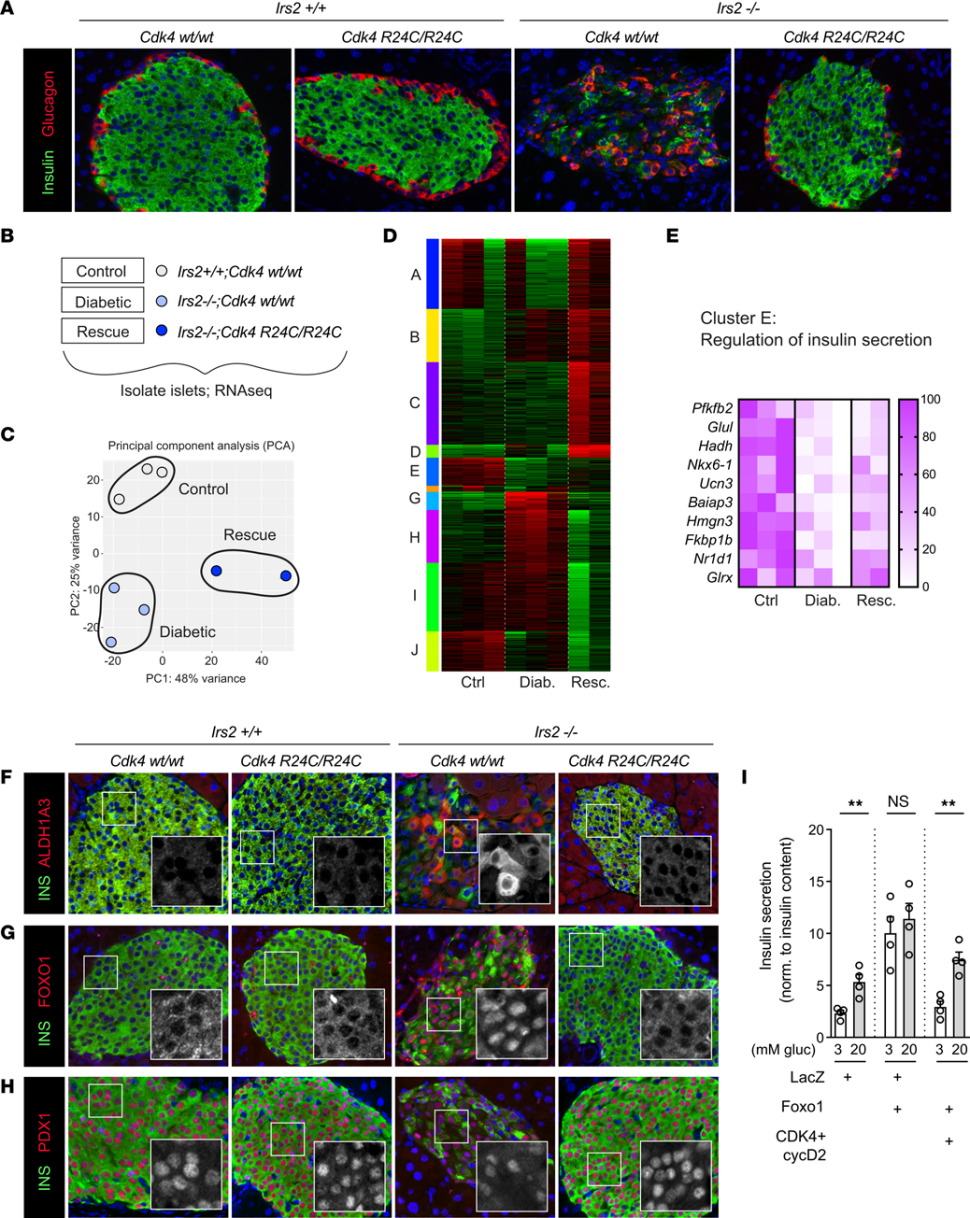
*Cdk4-R24C* corrected β **cell dedifferentiation in** ***Irs2^–/–^*** **islets.** (**A**) Pancreas sections stained for insulin (green) and glucagon (red). (**B**–**E**) Bulk RNA-Seq of islets isolated from Control (*Irs2^+/+^*;*Cdk4*-*WT/WT) (n* = *3)*, Diabetic *(Irs2*^–/–^;*Cdk4*-*WT/WT) (n* = *3)*, and Rescue (*Irs2*^–/–^;*Cdk4*-*R24C/R24C)* (*n* = 2) genotypes. Principal component analysis showed clean partitioning of the genotypes (**C**). K-means clustering (**D**) revealed 3 clusters in which mRNA abundance was disrupted during decompensation (Diabetic) and partially recovered (Rescue). Cluster E, containing genes that were reduced in *Irs2*^–/–^; *Cdk4*-*WT/WT* (Diabetic) mice and partially restored in *Irs2*^–/–^;*Cdk4*-*R24C/R24C* (Rescue) mice, shown in the heat map in (**E**) mapped to the GO term regulation of insulin secretion. (**F**–**H**) Pancreas sections immunostained for insulin (green), dapi (blue), and ALDH1A3 (**F**), FOXO1 (**G**), or PDX1 (**H**) (all red) confirm that β cell dedifferentiation signature in *Irs2^–/–^* male mice was rescued by homozygous *Cdk4-R24C*. Grayscale insets reflect red-channel immunofluorescence in (**F**–**H**). (**I**) Glucose stimulated insulin secretion performed on dispersed mouse islet cells transduced with the indicated adenoviruses, normalized to insulin content (*n* = 4). Images in **A** and **F**–**H** are representative of *n* ≥ 2 experiments. Original magnification, ×200(**A** and **F**–**H**). Statistics by *t* test; **P < 0.01.

Although proliferation was statistically increased in male *Irs2^–/–^;Cdk4-R24C/R24C* mice compared with *Irs2^–/–^* controls, the overall frequency of proliferation remained low and seemed insufficient to explain the marked improvement in islet morphology and insulin content. To better assess islet architecture, we performed immunofluorescence for insulin and glucagon (Figure 3A). *Irs2^–/–^;Cdk4-WT/WT* islets from male mice stained poorly for insulin, with reduced staining intensity in general and marked heterogeneity among β cells compared with controls. Islet architecture was also disrupted in male *Irs2^–/–^* islets, with α cells no longer restricted to the periphery but instead intermixed with β cells (Figure 3A). Many of the insulin-low cells were also negative for glucagon, suggesting impaired β cell insulin production rather than cell loss with resulting islet collapse, consistent with the previously demonstrated β cell dedifferentiation reported in this model (14, 28). Remarkably, the quality and intensity of insulin staining, and the islet architecture, were completely restored to normal levels in male mice bearing homozygous *Cdk4-R24C* alleles (Figure 3A).

### Gene signature analysis suggested rescue of β cell function.

To dissect the mechanism by which *R24C-R24C* rescued Irs2^–/–^ islet function, we used RNA-Seq to assess gene expression level changes. Whole islets were isolated from adult male mice of control (*Irs2^+/+^*;*Cdk4*-*WT/WT)*, diabetic *(Irs2*^–/–^;*Cdk4*-*WT/WT)*, and rescue (*Irs2*^–/–^;*Cdk4*-*R24C/R24C)* genotypes (Figure 3B) and sent for library generation and sequencing. The samples cleanly partitioned based on principal component analysis (Figure 3C). Intriguingly, the major GO terms determining sample partitioning in principal components 1 and 2 involved RNA processing and peptide translation (Supplemental Figure 4A). Differential expression analysis revealed a number of differences between diabetic islets and controls (566 genes up, 327 genes down) and between rescue islets and diabetic islets (1,064 up, 1,115 down) (Supplemental Figure 4, B–D). K-means clustering identified genes for which expression was reduced in diabetic islets and regained in rescue, or expression was increased in diabetic islets and restored to normal levels in rescue. Three clusters (E, G, H) matched these patterns (Figure 3D and Supplemental Table 1). GO term mapping of genes increased in diabetic islets and corrected in rescue islets (clusters G–H) included protein glycosylation and response to ER stress. Cluster E, which contains genes lost in diabetic islets and partially regained in *R24C/R24C* rescued islets, mapped to the GO term regulation of insulin secretion (Figure 3E and Supplemental Table 2). Genes in cluster E included several genes with key roles in insulin secretion including β cell maturation factors *Nkx6–1* and *Ucn3*, regulators of *Slc2a2* expression (*Hmgn3*), glycolysis and GK localization (*Pfkfb2*), mitochondrial metabolism (*Glul*, *Hadh*), dense-core granule biogenesis and maturation (*Baiap3*), and NADPH regulation of exocytosis (*Glrx*) (29–34). These data, though weakened by the caveats of bulk sequencing, inhomogeneous cell populations, and differences in the metabolic environment from which the samples were taken, supported the histological impression that *R24C/R24C* might improve β cell function in *Irs2^–/–^* male mice (14, 28).

### Cdk4-R24C corrected β cell dedifferentiation in IRS2 null male mice.

To assess dedifferentiation, we stained pancreas sections from male mice for aldehyde dehydrogenase 1 isoform A3 (ALDH1A3), a marker of failing β cells (35). Intriguingly, the strong ALDH1A3 labeling observed in *Irs2*^–/–^;*Cdk4-WT/WT* β cells was corrected in *Irs2^–/–^; Cdk4-R24C/R24C* mice (Figure 3F). Similarly, rare islet cells that stained for both insulin and glucagon were observed in *Irs2*^–/–^;*Cdk4-WT/WT* pancreas sections; these double-positive cells were not evident in *Irs2^–/–^; Cdk4-R24C/R24C* mice, suggesting rescue of this phenotype as well (Supplemental Figure 5). FOXO1, a transcription factor inhibited by insulin signaling, is active in IRS2-null mice and drives β cell failure (14, 28). We hypothesized that *CDK4-R24C* might prevent FOXO1 activation in this model, mitigating the deleterious FOXO1 suppression of *Pdx1* expression. To investigate whether the FOXO1-PDX1 axis was restored in *Irs2*^–/–^; *Cdk4-R24C/R24C* islets, we first stained for FOXO1. Confirming prior observations (14), FOXO1 immunostaining was abnormally localized in β cell nuclei in *Irs2^–/–^; Cdk4-WT/WT* islets (Figure 3G and Supplemental Figure 6), consistent with reduced AKT-mediated phosphorylation of FOXO1 in the absence of IRS2. As previously published, *Irs2^–/–^; Cdk4-WT/WT* islets also had reduced nuclear staining for PDX1 (Figure 3H). Intriguingly, *Cdk4-R24C* homozygosity restored both FOXO1 and PDX1 staining to WT appearance, with FOXO1 restricted to the cytoplasm and PDX1 robustly nuclear (Figure 3, G and H). With the caveat that some of the dedifferentiation rescue may have been due to correction of the hyperglycemic environment in *Irs2^–/–^* mice, this result suggests a role for activated CDK4 in promoting distal insulin signaling in the β cell, specifically in protecting against FOXO1-mediated β cell dedifferentiation in the setting of IRS2 deficiency in male mice.

To test definitively whether CDK4 can promote β cell function in the face of aberrant FOXO1 activity in an ex vivo setting where ambient glucose can be controlled, we assessed glucose-stimulated insulin secretion in dispersed mouse islet cells overexpressing FOXO1 with or without CDK4 in combination with its activator cyclin D2 (Figure 3I). Overexpression of FOXO1 increased insulin secretion under basal glucose conditions, showing failure to suppress in low glucose, and eliminated the increase in high glucose, consistent with impaired β cell function. Remarkably, cotransducing the islet cell cultures with CDK4/cyclinD2 restored both the suppression of insulin release in low glucose as well as the increase induced by a switch to high glucose. These data support the concept that CDK4 has protective activity against FOXO1-mediated β cell dedifferentiation.

### CDK4 rescued Pdx1 and Neurod1 expression in starve conditions ex vivo.

**Figure 4:**
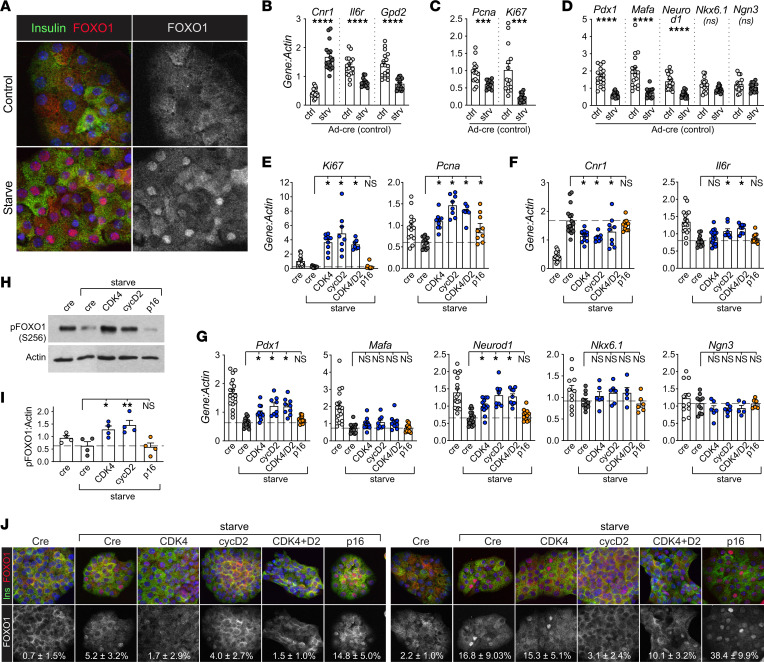
CDK4 suppresses starvation-induced FOXO1 activity in mouse islet cells. (**A**) Dispersed mouse islet cells cultured in islet complete medium (15 mM glucose, 10% FBS, control) or starve medium (2 mM glucose, 0% FBS) for 16 hours were fixed, immunostained for FOXO1 (red), insulin (green), and Dapi (blue), and imaged by confocal microscopy. (**B**–**G**) Dispersed mouse islet cells transduced with the indicated adenoviruses were cultured as in **A**, then lysed for qPCR for known FOXO1 target genes (*n* ≥ 12) (**B**), proliferation markers (*n* ≥ 12) (**C**), and β cell differentiation genes (*n* ≥ 12) (**D**). (**E**–**G**) Transduction with CDK4 activators (Ad-h-CDK4, Ad-m-cyclinD2, and Ad-h-Cdk4+Ad-m-cyclinD2), or CDK4 inhibitor (Ad-m-p16) showed that activating CDK4 rescued abundance of proliferation markers (*n* ≥ 7) (**E**), rescued 2 of 3 FOXO1 targets (*n* ≥ 8) (**F**), and rescued *Pdx1* (*n* ≥ 8) and *Neurod1* (*n* ≥ 8) expression but not *Mafa* (*n* ≥ 8). *Nkx6.1* (*n* ≥ 5) and *Ngn3* (*n* ≥ 5) were not changed by starvation or CDK4 overexpression (**G**). Data in **B**–**D** are the controls from **E**–**G**, presented separately for clarity. (**H** and **I**) (*n* ≥ 4) Ad-h-CDK4 or Ad-m-cyclinD2 increased phosphorylation of FOXO1 in dispersed mouse islet cells exposed to starve conditions (**H**), but Ad-m-p16 did not, quantified in (**I**). Confocal microscopy (**J**) with quantification of the percentage β cells with nuclear FOXO1 showed nuclear FOXO1 was variably reduced by CDK4 activation. Top and bottom panels show 2 different biological replicates illustrating variability of nuclear FOXO1 abundance. Grayscale panels represent red-channel (FOXO1) immunofluorescence. Dashed lines represent mean starve control condition. Images in **A** and **J** are representative of *n* ≥ 4 experiments. Original magnification, ×400 (**A** and **J**). Statistics by *t* test (**B-D**) or 1-way ANOVA (**E**–**I**) with Tukey’s posthoc test. *P < 0.05; **P < 0.01; ***P < 0.001; ****P < 0.0001.

We developed an ex vivo mouse islet cell starvation model to test the effect of CDK4 on FOXO1 activation. For these studies, CDK4 activity was increased by overexpression of WT CDK4, cyclin D2, or both. Short-term (16 hour) starvation of dispersed islet cells led to a heterogeneous, but consistent, increase in nuclear FOXO1 (Figure 4A) and gene expression changes consistent with active nuclear FOXO1, such as increased *Cnr1* and decreased *Il6r* and *Gpd2* (Figure 4B) (36). Proliferation markers *PCNA* and *Ki67* were both decreased with starvation (Figure 4C). Starvation suppressed *Pdx1* expression, mimicking in vivo *Irs2* deletion; other β cell differentiation markers decreased (i.e., *Mafa* and *NeuroD1*) or were not affected (i.e., *Nkx6.1* and *Ngn3*; Figure 4D).

Using this starvation model, we tested whether CDK4 activation rescued the gene expression changes associated with nuclear FOXO1, by overexpressing CDK4 individually or in combination with cyclin D2. As a negative control, we overexpressed p16, an INK-family CDK4 inhibitor. As expected, the cell cycle activators increased proliferation markers *Ki67* and *Pcna*, while the inhibitor did not (Figure 4E). Interestingly, activating CDK4 partially rescued the starve-induced changes in 2 out of 3 FOXO1 targets (Figure 4F; *Gpd2* not rescued, not shown). Remarkably, overexpression of CDK4 with or without cyclin D2 rescued *Pdx1* and *Neurod1* mRNA abundance (Figure 4G); *Mafa* levels were not rescued. On the other hand, the p16 CDK inhibitor did not rescue the FOXO1 target genes (Figure 4F), nor *Pdx1* or *Neurod1* (Figure 4G). *Nkx6.1* and *Ngn3*, which were not reduced by starvation (Figure 4D), were also not impacted by any of the cell cycle regulator combinations.

Since the immunofluorescence and mRNA data suggested that CDK4 inhibited FOXO1 activity, we tested whether CDK4 overexpression increased FOXO1 phosphorylation. By immunoblot, starvation decreased FOXO1 phosphorylation at S256, while overexpression of CDK4 or cyclin D2, but not p16, restored FOXO1 phosphorylation (Figure 4, H and I). We hypothesized that CDK4 might directly phosphorylate FOXO1. Posttranslational modification prediction software (GPS 2.1) (37) predicted CDK4 consensus phosphorylation sites in FOXO1, some of which overlap with known AKT phosphorylation sites (data not shown). However, experiments using the AKT inhibitor MK-2206 showed that the increase in p-FOXO1 after CDK4 overexpression was dependent on AKT activation, suggesting an indirect mechanism. (Supplemental Figure 7).

### FOXO1 phosphorylation and activity did not correlate with subcellular localization.

We hypothesized that CDK4-mediated alleviation of FOXO1 *Pdx1* suppression was related to the FOXO1 nuclear exclusion reported to follow phosphorylation (38, 39). We first tested whether increased FOXO1 phosphorylation resulted in FOXO1 cytoplasmic localization. However, in these experiments confocal microscopy showed that starvation (dephosphorylated FOXO1) caused only heterogeneously nuclear β cell FOXO1 (Figure 4J). Further, overexpression of CDK4, which did increase FOXO1 phosphorylation (Figure 4, H and I), only variably decreased the percentage of cells with visibly nuclear FOXO1 (Figure 4J). Intriguingly, p16 overexpression consistently increased the percentage of β cells with nuclear FOXO1 in this starvation model.

The variability in visibly nuclear FOXO1 from one experiment to the next, and the failure of CDK4 to consistently redistribute FOXO1 to the cytoplasm, suggested the possibility that CDK4 rescue of FOXO1-mediated *Pdx1* suppression might not require changing FOXO1 subcellular localization. To further explore the relationship between FOXO1 localization and expression of its transcription targets, we tested whether relocalizing FOXO1 to nuclei was sufficient to increase FOXO1 target gene expression. First, we treated mouse islet cells cultured in 15 mM glucose with the nuclear export inhibitor Leptomycin B (40). Surprisingly, even in high glucose conditions where insulin signaling is fully activated in β cells (15, 41), FOXO1 rapidly accumulated in the nucleus within 5–30 minutes of treatment with Leptomycin B, suggesting FOXO1 shuttles between the cytoplasm and nucleus even in nutrient replete conditions (Supplemental Figure 8A). FOXO1 targets *Cnr1* and *Il6r* changed abundance in the expected direction; *Gpd2*, on the other hand, increased (Supplemental Figure 8B). Despite robust nuclear accumulation of FOXO1, FOXO1 targets *Pdx1* and *Neurod1* did not decrease, but rather significantly increased (Supplemental Figure 8C).

**Figure 5:**
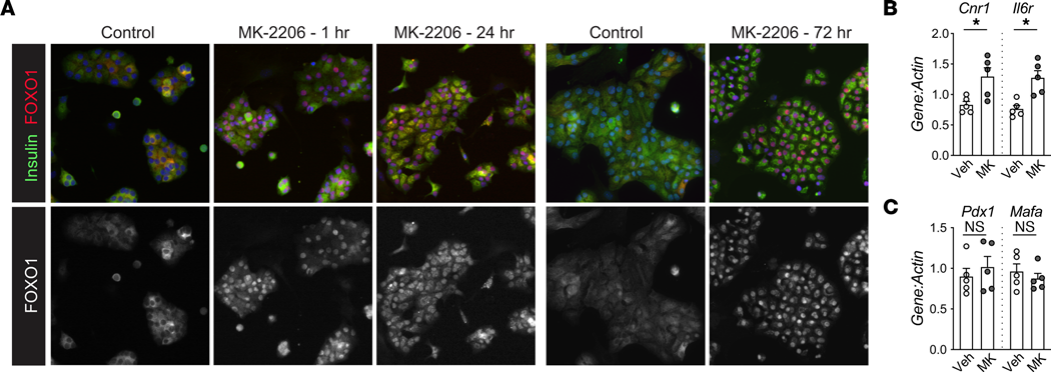
Forced nuclear accumulation of FOXO1 is not sufficient to repress *Pdx1*. (**A**–**C**) Dispersed mouse islet cells cultured in ICM with 15 mM glucose were treated with AKT inhibitor MK-2206 (5 uM) for the indicated durations, then fixed, immunostained for insulin (green), FOXO1 (red), and DAPI (blue), and imaged by fluorescence microscopy, with the red (FOXO1) channel displayed separately below in grayscale (**A**) or lysed for qPCR (72 h) to measure FOXO1 target gene expression (*n* = 5) (**B**) or β cell maturation genes (*n* = 5) (**C**). Images in **A** are representative of *n* = 2 experiments. Original magnification, ×200 (**A**). Statistics are by unpaired *t* test. *P < 0.05.

Leptomycin B traps many factors in the nucleus, so these effects may be independent of FOXO1. To more specifically test the relationship between nuclear FOXO1 and *Pdx1* suppression, we treated dispersed islet cells with MK-2206, a pan-AKT inhibitor (42). We expected that inhibiting the kinase primarily responsible for phosphorylating FOXO1 (38) would lead to nuclear FOXO1 and suppression of *Pdx1*. Indeed, inhibiting AKT led to rapid and sustained FOXO1 nuclear accumulation (Figure 5A). However, despite nuclear localization, FOXO1 failed to suppress *Pdx1* or elicit other expected gene expression changes (Figure 5B-C). With the caveat that both of these approaches alter cellular biology beyond FOXO1 modulation, these results suggested that nuclear localization of FOXO1 was not sufficient to suppress *Pdx1* in mouse β cells, echoing the lack of correlation between FOXO1 nuclear localization and transcription regulation described in other cell systems (43, 44).

### CDK4 rescue of FOXO1-induced Pdx1 repression is independent of FOXO1 phosphorylation or subcellular localization.

**Figure 6:**
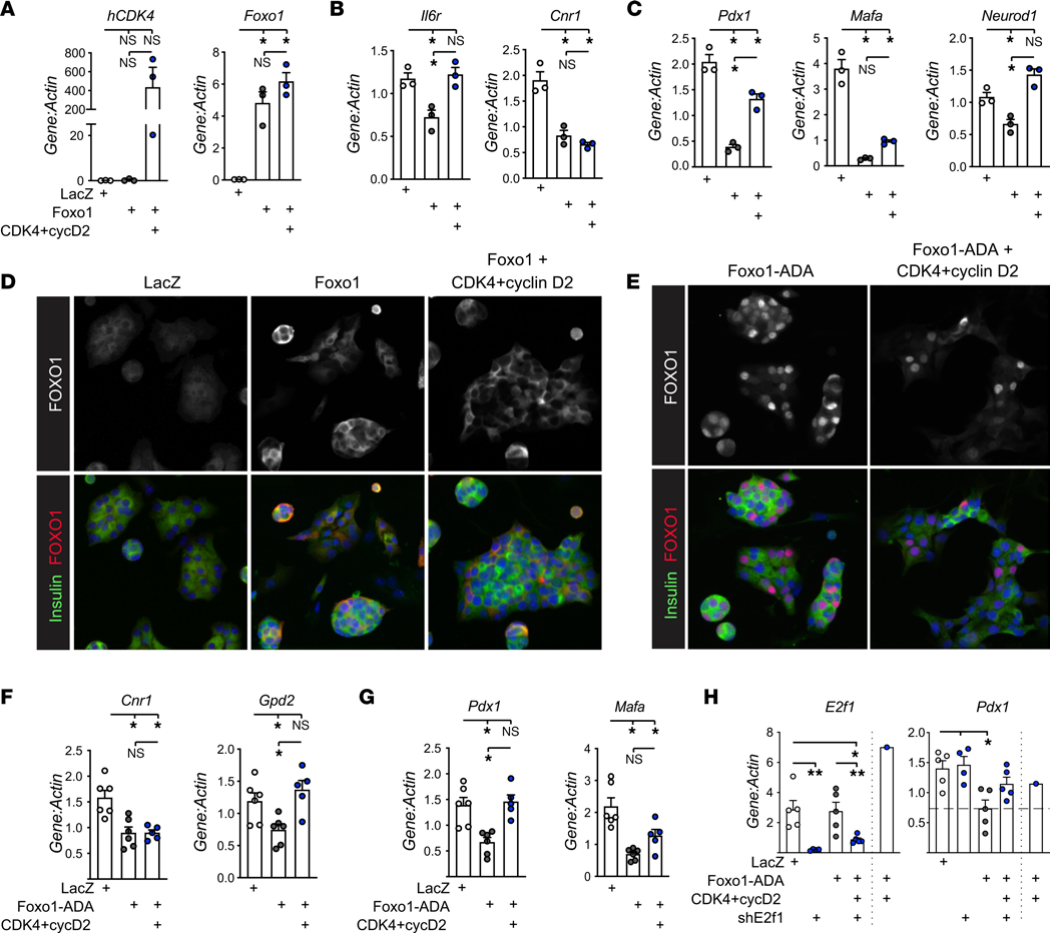
CDK4 rescues FOXO1-mediated *Pdx1* suppression even with an unphosphorylatable FOXO1-ADA mutant. (**A**–**D**) Dispersed mouse islet cells were transduced with Ad-LacZ control, Ad-m-Foxo1, and/or Ad-h-CDK4 with Ad-m-cyclin D2, cultured for 72 hours in ICM with 15 mM glucose, then analyzed by qPCR for (**A**) *hCDK4* or *Foxo1* to assess transduction (*n* = 3), (**B**) FOXO1 targets (*n* = 3), or (**C**) β cell differentiation genes (*n* = 3); (**D**) parallel cultures on glass coverslips were immunostained for insulin (green), FOXO1 (red) and DAPI (blue) to assess FOXO1 localization in β cells. (**E**–**G**) Dispersed mouse islet cells were transduced with Ad-LacZ (control), Ad-m-FOXO1-ADA (unphosphorylatable mutant), and Ad-h-CDK4 + Ad-m-cyclinD2, cultured for 72 hours in ICM with 15 mM glucose, and subjected to (**E**) immunostaining for insulin (green), FOXO1 (red), and DAPI (blue) to assess FOXO1 localization in β cells, or qPCR for (**F**) FOXO1 targets (*n* ≥ 5) or (**G**) β cell differentiation genes (*n* ≥ 5). (**H**) Dispersed mouse islet cells transduced with the indicated adenoviruses for 72 hours in ICM with 15 mM glucose were lysed for qPCR for *E2f1* (*n* ≥ 4) or *Pdx1* (*n* ≥ 4). CDK4+CycD2 (*n* = 1) is shown for comparison but not included in the statistical analysis. Images in **D** and **E** are representative of *n* ≥ 2 experiments. Original magnification, ×200 (**D** and **E**). Statistics are by 1-way ANOVA with Tukey’s posthoc test, *P < 0.05.

Lack of influence of CDK4 on FOXO1 localization led us to question whether CDK4 derepression of *Pdx1* involved FOXO1. To directly test whether CDK4 could counteract FOXO1-mediated *Pdx1* suppression we overexpressed FOXO1 in dispersed mouse islet cells cultured in high glucose and examined the impact of CDK4 coexpression (Figure 6A). FOXO1 overexpression decreased *Il6r* and *Cnr1*, both known FOXO1 targets (Figure 6B). Consistent with prior reports, FOXO1 overexpression suppressed *Pdx1* mRNA (Figure 6C); different from prior reports, *MafA* and *Neurod1* were also decreased (14, 36, 45). Coexpression of CDK4 and cyclin D2 led to rescue of *Il6r* but not *Cnr1*, and, intriguingly, resulted in partial or complete rescue of *Pdx1*, *Mafa*, and *Neurod1* (Figure 6C), confirming that CDK4 inhibited FOXO1-mediated repression of β cell maturation factors. Despite overexpression, FOXO1 did not visibly accumulate in β cell nuclei (Figure 6D), reconfirming the lack of correlation between nuclear FOXO1 localization and gene expression changes in this system.

To test whether phosphorylation of FOXO1 was required for CDK4-mediated derepression, we expressed the FOXO1-ADA mutant. FOXO1-ADA has serine to alanine/aspartic acid mutations in 3 AKT phosphorylation sites (T24A, S253D, and S316A), making FOXO1 resistant to AKT phosphorylation, and, thus, constitutively active and nuclear (46, 47). As expected, expressing FOXO1-ADA in dispersed mouse islet cells resulted in nuclear FOXO1 labeling (Figure 6E). FOXO1-ADA repressed FOXO1 targets *Cnr1* and *Gpd2* (Figure 6F) as well as *Pdx1* and *Mafa* (36) (Figure 6G). Surprisingly, coexpression of CDK4+cyclinD2 partially or completely restored expression of FOXO1 target *Gpd2* (Figure 6F), as well as *Pdx1* and *Mafa*, suggesting that CDK4-mediated derepression of *Pdx1* does not require FOXO1 phosphorylation at AKT sites (Figure 6G). *Cnr1*, which was not rescued by CDK4+cyclinD2 in the context of WT-FOXO1 (Figure 6B), was also not rescued in the setting of FOXO1-ADA (Figure 6F). We explored whether CDK4 derepression of *Pdx1* expression involved canonical CDK activity via Rb phosphorylation and increased E2F1 activity, but found that knockdown of *E2f1* did not prevent CDK4 rescue of FOXO1-ADA mediated *Pdx1* suppression (Figure 6H). Taken together, these experiments suggest that although CDK4 overexpression increased FOXO1 phosphorylation in AKT-dependent fashion, the CDK4-mediated inhibition of FOXO1 repression of *Pdx1* and *Mafa* were independent of FOXO1 phosphorylation, nuclear localization, or E2F1 activation.

### CDK4 may modulate FOXO1 activity through deacetylation and promotion of FOXO1 degradation.

**Figure 7:**
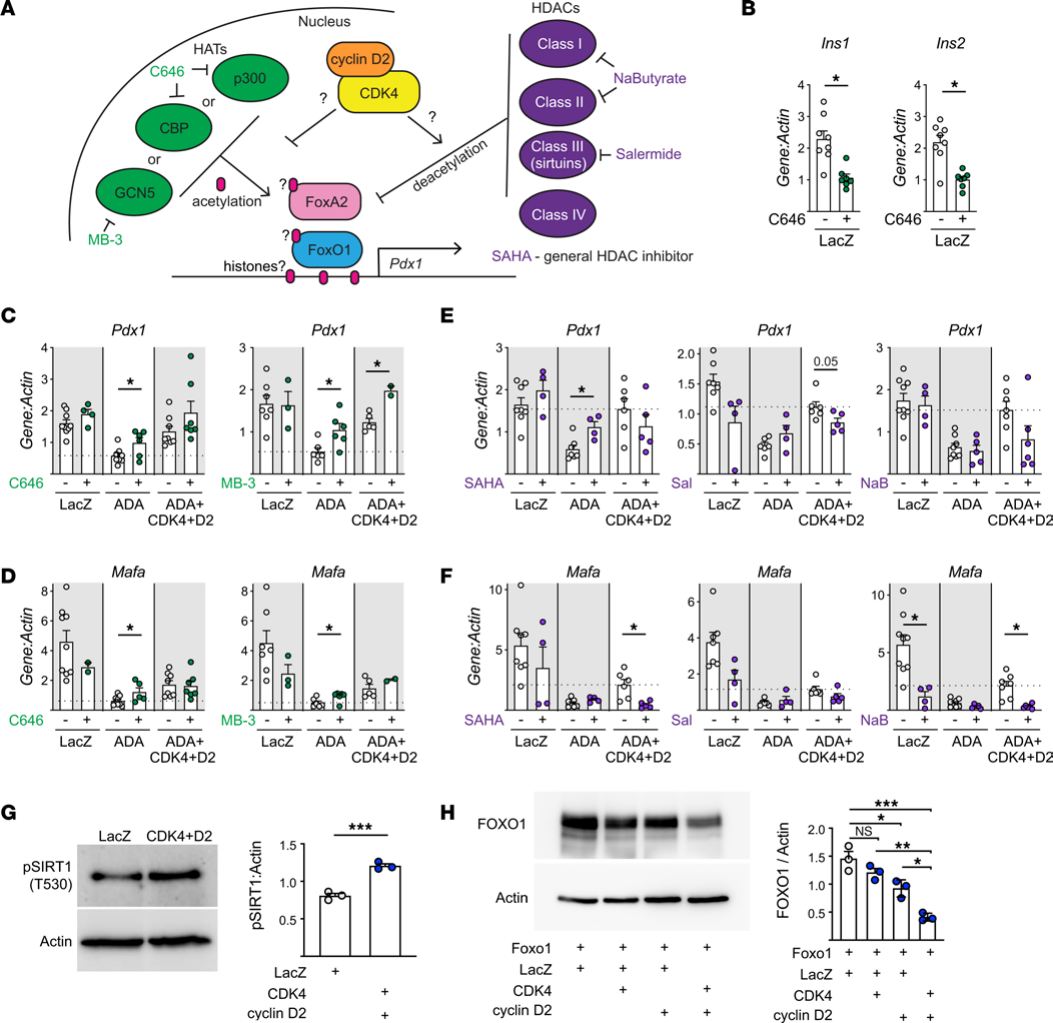
CDK4 rescue of FOXO1 *Pdx1* and *Mafa* suppression is modulated by acetylation. (**A**) Model expressing the hypothesis that CDK4 influences acetylation of key players at the *Pdx1* locus, with diagram of HATs and their inhibitors (green) and HDACs and their inhibitors (purple). (**B**–**F**) Dispersed mouse islet cells were transduced with the indicated adenoviruses, then cultured for 72 hours with or without the indicated HAT (**B**–**D**) (*n* = 2–9) or HDAC (**E** and **F**) (*n* = 4–9) inhibitors, then analyzed by qPCR for *Pdx1* or *Mafa*. (**G**–**H**) (*n* = 3) Dispersed mouse islet cells were transduced with the indicated adenoviruses, cultured for 72 hours in ICM with 15 mM glucose, then lysed and analyzed by immunoblot. Statistics are by 1-way ANOVA with Tukey’s posthoc test (**B**–**F** and **H**) or by unpaired *t* test (**G**). *P < 0.05, **P < 0.01, ***P < 0.001.

**Figure 8:**
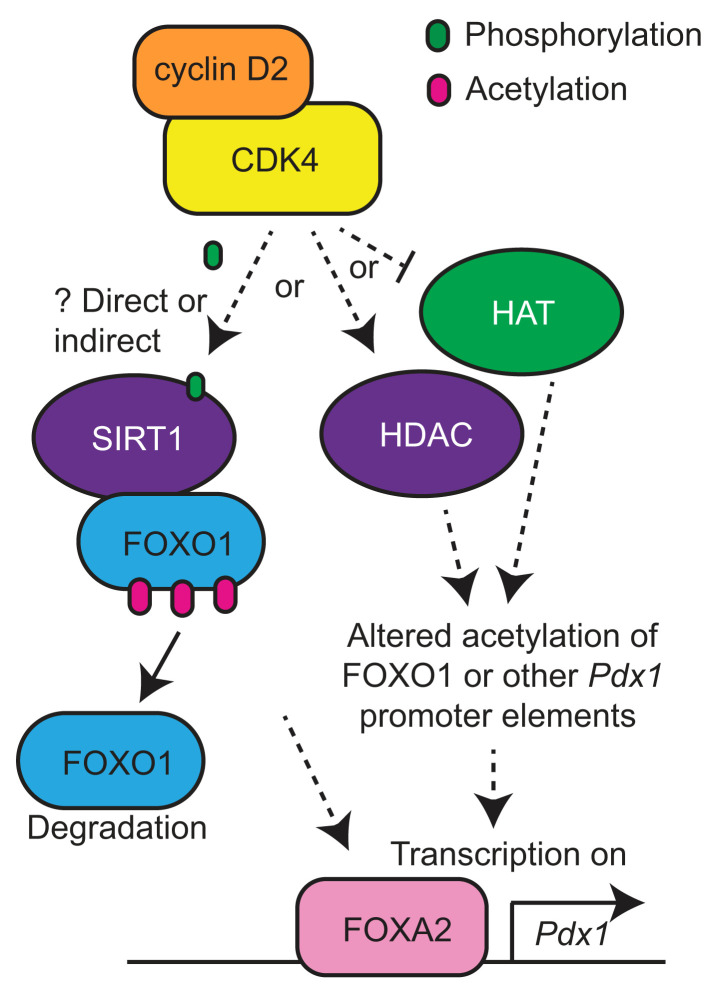
Model of how CDK4 may rescue FOXO1-mediated suppression of *Pdx1* transcription through deacetylation. Activation of the CDK4 kinase results in increased phosphorylation of the SIRT1 deacetylase, either through direct phosphorylation or indirectly, which may deacetylate FOXO1, resulting in degradation of FOXO1 and derepression of *Pdx1*. Alternatively or in addition, CDK4 may directly or indirectly inhibit HAT activity or promote activity of HDACs other than SIRT1 to derepress *Pdx1* gene transcription.

FOXO1 activity is regulated not only by phosphorylation but also by acetylation (45–50). Previous reports show CDKs can activate both HATs (27) and HDACs (51, 52). We tested whether modulating HAT or HDAC activity impacted FOXO1 suppression of *Pdx1* using small molecule inhibitors of HATs (MB-3 to inhibit GCN5 and C646 to inhibit p300/CBP) and HDACs (salermide to inhibit the sirtuins, sodium butyrate to inhibit class I/II HDACs, and vorinostat/SAHA as a general HDAC inhibitor) (Figure 7A). As previously demonstrated (53, 54), inhibiting p300 reduced *Ins1* and *Ins2* abundance (Figure 7B). Using the FOXO1-ADA mutant to eliminate influences on FOXO1 phosphorylation and localization, we found that inhibiting HATs tended to derepress *Pdx1* and *Mafa* to a similar degree as overexpression of CDK4/cyclin D2 (Figure 7, C and D). Conversely, inhibiting HDACs tended to have the opposite effect, impairing CDK4/cyclin D2-induced derepression of *Pdx1* and *Mafa* (Figure 7, E and F). These results suggest that CDK4/cyclin D2 acted to either activate HDAC activity or repress HAT activity. Deacetylated FOXO1 had enhanced transcriptional activity (55, 56) but also increased degradation (45). Intriguingly, overexpression of CDK4/cyclinD2 increased phosphorylation of SIRT1, known to deacetylate FOXO1 in β cells (45) and FOXO family members in other cell types (57–59) (Figure 7G). We attempted to assess FOXO1 acetylation status by immunoprecipitation but were unable to confidently quantify this parameter (data not shown). However, we did observe that CDK4 and cyclin D2 decreased total FOXO1 protein levels, especially when expressed in combination, consistent with enhanced degradation (Figure 7H). That said, we did not experimentally confirm a role for SIRT1 in this system; the observed changes in SIRT1 phosphorylation may be unrelated to changes in FOXO1 activity and *Pdx1* expression. In summary, these results suggest that CDK4 impacted acetylation status either of FOXO1 itself or other elements at the *Pdx1* promoter, via either decreased HAT activity or increased HDAC activity, to derepress FOXO1-induced *Pdx1* suppression and reduce FOXO1 abundance (Figure 8).

## Discussion

This work identifies a surprising role for the cell cycle activator CDK4 in the modulation of insulin signaling in the adult pancreatic β cell, counteracting FOXO1-mediated dedifferentiation. Replacing both endogenous alleles of *Cdk4* with *Cdk4-R24C* prevented diabetes in IRS2-null male mice by rescuing β cell mass, function, and differentiation and restoring cytoplasmic FOXO1 location and nuclear PDX1 abundance in β cells in vivo. Ex vivo, CDK4 rescued FOXO1 impairment of insulin secretory function and inhibited FOXO1 suppression of *Pdx1* through 2 distinct mechanisms: AKT-dependent phosphorylation of FOXO1 and a second mechanism involving either reduced acetylation or increased deacetylation, with possible involvement of SIRT1. Together, these results demonstrate an unexpected role for CDK4 not only in driving the cell cycle but also in promoting β cell differentiation and function.

The most important finding from these studies is that CDK4 can promote or maintain β cell differentiation. It remains controversial whether β cells must dedifferentiate to proliferate (11, 60). Replacing both alleles of *Cdk4-WT* with *Cdk4-R24C* rescued not only β cell proliferation but also dedifferentiation in diabetic IRS2-null mice, supporting reports that it is possible to increase β cell proliferation without negatively impacting β cell function (60–66). A related observation in neurons was recently published (67). Since the primary source of new β cells in the adult mouse is replication of mature β cells (68, 69), the mutual incompatibility of proliferation and differentiation described in fetal development and adult stem cell activation paradigms may not apply. Indeed, CDKs are implicated in endocrine development (70, 71). Taken in this context, the observation that CDK4, a critical cell cycle driver in endocrine cells, can also promote their differentiation may represent a key piece in our understanding of adult islet homeostasis.

The degree of rescue of dedifferentiation by homozygous *CDK4-R24C* observed in this study was surprisingly complete, especially given the lack of improvement in insulin resistance, suggesting that loss of CDK4/cyclinD2 activity may be a primary cause of β cell failure in IRS2-null mice. The IRS2-null phenotype was only partially rescued by FOXO1 haplosufficiency (14) or transgenic overexpression of *Pdx1* (28). *CDK4-R24C* may have additional parallel benefits such as bolstering β cell mass through proliferation or effects on lysosomal and mitochondrial biology (72). Our result is consistent with a prior observation that overexpression of CDK4-R24C under the insulin promoter rescued diabetes due to leptin receptor deficiency (73). That result was attributed to expansion of β cell mass rather than a direct effect on β cell differentiation. Other studies have also noted the surprising maintenance of physiological function of β cells driven to proliferate by CDK4 activation (25).

These results reconfirm the known role for CDK4 in promoting β cell proliferation (21, 22, 25). Insulin resistance in *Irs2^–/–^;Cdk4-R24C/R24C* mice likely contributed to increased proliferation (74, 75). Cyclin D2 drives β cell compensation in response to insulin resistance (20), but islet cyclin D2 levels are reduced in *Irs2^–/–^* mice (15); rescue by *Cdk4-R24C* makes sense in this context. Residue 24 in CDK4 not only mediates INK-family inhibition but also D-cyclin binding (76), so CDK4-R24C may have reduced regulatory inputs in both directions.

Proliferation was not statistically increased in *Irs2^+/+^;Cdk4-R24C/R24C* mice; increased β cell mass in this group could be due to increased proliferation at an earlier time point. Alternatively, *Cdk4-R24C* allele may have expanded the pool of Ngn3^+^ endocrine precursors to increase β cell mass in the adult (70, 71). Oddly, although proliferation was highest in *Irs2^–/–^; Cdk4-R24C/R24C* mice, β cell mass was not higher in this group relative to *Irs2^+/+^;Cdk4-R24C/R24C* mice. Since *Irs2^–/–^;Cdk4-R24C/R24C* mice are insulin resistant with elevated fat mass, we postulated that that group might have higher β cell apoptosis, but β cell death was, if anything, reduced in those mice. It is possible they had competing suppression of proliferation by elevated free fatty acids (17). Finally, given the hyperinsulinemia in response to insulin resistance in *Irs2^–/–^;Cdk4-R24C/R24C*, CDK4 may synergize with activation of the unfolded protein response to drive β cell proliferation (77).

Chronic hyperglycemia in IRS2-null mice may contribute to dedifferentiation via oxidative stress or glucotoxicity, but it is likely not the only cause, since *Pdx1* expression is reduced before the onset of hyperglycemia (28). Additionally, CDK4 promoted FOXO1 phosphorylation and increased *Pdx1* ex vivo in islet cells when glucose levels were held constant (Figures 4, 6 and 7). Surprisingly, *MafA* and *NeuroD1* were not induced by FOXO1 as previously reported (45), which suggests that FOXO1 targets may be sensitive to cellular context or type of stress (78).

These data suggest a role for INK family cell cycle inhibitors in β cell dedifferentiation. Although the *Cdk4-R24C* allele has been termed constitutively active, the behavior of this mutant in our study was more consistent with either a weak gain of function requiring 2 alleles for measurable impact, or potentially a loss-of-function mechanism, since the heterozygous state resembled controls and rescue was observed only in the homozygous state. Similarly, Rb phosphorylation was not always increased by *Cdk4*-*R24C* in the heterozygous state (79). A possible explanation is that this phenotype is dependent on loss of INK-family inhibition of CDK4 rather than activation of CDK4 kinase activity. Interestingly, p27, a Cip/Kip cell cycle inhibitor, is a cause of β cell failure in IRS2-null mice (80). The *R24C* mutation prevents INK4-family CDK4 inhibition (23, 24); in cancers, *Cdk4-R24C* mutation is synergistic with loss of function of Cip/Kip inhibitors (79), suggesting that *Cdk4-R24C* may counteract a damaging combination of loss of cyclin D2 with gain of p27 in IRS2 null β cells.

Surprisingly, *CDK4-R24C* did not rescue insulin resistance in other metabolic tissues. CDK4 improves insulin sensitivity through PPARγ activation in adipocytes (26) and suppression of hepatic glucose production (27). Fat mass was increased in *Irs2^–/–^; Cdk4-R24C* mice, although this could have been secondary to hyperinsulinemia. In our study, fat mass was not increased in *Irs2^+/+^; Cdk4-R24C* mice.

Our data support a model in which FOXO1 subcellular localization is not the sole input into regulation of its gene targets. We initially expected that conditions in which FOXO1 immunostaining showed cytoplasmic localization would not show suppression of *Pdx1* mRNA, while nuclear FOXO1 would correlate with repressed *Pdx1* and other related changes in known FOXO1 targets (14, 36, 38, 45, 47). Our data, at times, did not support this model. Inhibition of nuclear export using leptomycin showed that even in glucose-excess conditions, FOXO1 passed through the nucleus. Despite forced retention of FOXO1 in the nucleus using leptomycin or AKT inhibition, *Pdx1* mRNA was not suppressed. Together, these experiments suggest that predominant nuclear localization of FOXO1 was not sufficient to suppress *Pdx1*, and that regulation of subcellular localization and transcriptional activity of FOXO1 was not as straightforward as the phosphorylated form being cytoplasmic and off, while the nuclear form is on and repressing *Pdx1*. This more complicated relationship between FOXO1 localization and transcriptional activity is supported by prior studies in other systems (43, 44).

CDK4 increased phosphoryated FOXO1, but our data suggest that CDK4 does not directly phosphorylate FOXO1 in islet cells. Besides AKT, other kinases such as SGKs, DYRK1, and CDK2 can phosphorylate FOXO1 (78, 81–83); CDK2 phosphorylates FOXO1 at a critical serine residue and regulates its subcellular localization in a glucose-dependent manner (83). CDK4 rescue of *Pdx1* gene expression was lost in the presence of AKT inhibitor, suggesting, most likely, a requirement for AKT itself or for an AKT-dependent kinase. However, CDK4 rescued *Pdx1* expression even in the context of the unphosphorylatable FOXO1-ADA mutant (Figure 6), suggesting an additional mechanism besides phosphorylation.

CDK4 may regulate FOXO1 activity through acetylation or deacetylation, since HAT inhibitors potentiated and HDAC inhibitors suppressed the rescue (45, 55–57). Intriguingly, this result is different from the acetylation-promoting action of CDK4 observed in hepatocytes (27). The data are consistent with CDK4 reducing acetylation of transcription factors rather than histones, since acetylation of histones tends to promote gene expression due to increased access of transcription factors to DNA (84). In line with this, CDK4 increased the phosphorylation of SIRT1; sirtuins are the main class of HDACs that deacetylate FOXO1 (51, 52, 55, 57–59). However, we cannot conclude that the effects are through modulation of FOXO1 acetylation specifically, as we were not able to quantify acetylated FOXO1. FOXA2 promotes *Pdx1* expression (85) and can be deacetylated by SIRT1. FOXA2 deacetylation was reported to reduce its transcription activity in the liver and target it for degradation (86), but studies in the MIN6-β cell line found that SIRT1 deacetylated FOXA2 to promote *Pdx1* expression (87). It will be interesting to investigate whether FOXO1 or FOXA2 acetylation is impacted by CDK4 in future studies.

CyclinD2/CDK4 reduced FOXO1 protein abundance in cultured islet cells, consistent with accelerated degradation. While FOXO1 can be degraded after phosphorylation and cytoplasmic retention by 14-3-3 proteins (39, 88, 89), deacetylated FOXO1 is also more rapidly targeted for proteasomal degradation (45). Based on the observation that CDK4 promoted SIRT1 phosphorylation, we propose that CDK4/cyclinD2 increases the deacetylation of FOXO1 via SIRT1, which leads to its degradation and derepression of *Pdx1*. In the path toward β cell dedifferentiation, FOXO1 is nuclear during a metabolic inflexibility period (9, 90), but after exposure to chronic hyperglycemia, FOXO1 abundance declines (9). Since glucose increases cyclin D2 protein expression (1, 15, 18), we speculate that the degradation of FOXO1 observed in late-stage dedifferentiation is mediated by chronic glucose stimulation of these cell cycle regulators and increased HDAC activity (45).

Evidence supports a role for IRS2 in human diabetes. Polymorphisms at *IRS2* are associated with HBA1C and risk of T2D (91). *IRS2* variants are associated with glucose intolerance in women experiencing obesity (92), with diabetes-related traits in people of Pima ancestry (93), and with insulin resistance in people of Japanese ancestry with obesity (94). The Gly1057Asp *IRS2* polymorphism was (95) or was not (96) associated with impaired human β cell function. However, *IRS2* mRNA was reduced in islets from individuals with T2D relative to individuals in the control group (97), suggesting the possibility that a therapeutic intervention that promotes noncanonical CDK4 activity could have relevance in this scenario.

Strengths of this study include the use of parallel in vivo and ex vivo model systems to explore the fundamental biology of how a cell cycle regulator rescues diabetes phenotype in Irs2^–/–^ mice, a careful and comprehensive determination of the CDK4-mediated effect on FOXO1 biology and *Pdx1* expression, and the use of primary cells rather than transformed cell lines. Limitations of this study include the use of murine models and not human samples; future studies will be needed to demonstrate relevance to human β cells. The RNA-Seq was performed on whole islets rather than on single cells. We did not test FOXO1 occupancy at the *Pdx1* promoter by ChIP, which would shed further mechanistic light on how CDK4 impacts the molecular regulation of *Pdx1* mRNA abundance. Note that FOXO1 was previously shown to bind to the *Pdx1* promoter (14). Additional remaining questions include residual uncertainty as to which specific targets are phosphorylated by CDK4 to modulate the AKT node of the insulin signaling pathway and whether acetylation of FOXO1 itself is modulated. Given the increased use of CDK4 and 6 inhibitors in breast cancer, an important question is whether CDK4 inhibition impairs β cell function or differentiation.

In summary, we report the discovery that CDK4 promotes insulin signaling and FOXO1 degradation in the pancreatic β cell, derepressing *Pdx1* expression and rescuing diabetes in *Irs2^–/–^* mice. Our data suggest that CDK4 promotes both β cell proliferation and differentiation, suggesting that if safe therapeutic approaches can be developed to increase β cell mass via CDK4 they may preserve function.

## Methods

### Animal husbandry.

*Cdk4-R24C* mice, in which the *R24C* mutation is knocked in at the *Cdk4* locus (22), were obtained from the laboratory of Sushil Rane, National Institutes of Health, Bethesda, Maryland, USA, and were initially bred to *Irs2-*heterozygous mice (*B6;129-Irs2^tm1Mfw^/J*), which were rederived from cryopreserved stocks at The Jackson Laboratories, Bar Harbor, Maine, USA (12). Subsequently, mice heterozygous for both *Irs2* and *Cdk4-R24C* were crossed into to the C57BL/6N background, but not extensively backcrossed; C57BL/6N status was verified by *Nnt* genotyping. Littermate controls, using all relevant genotypes over multiple litters, were used for the in vivo metabolic studies, pancreas histology, and islet RNA-Seq. WT mice for ex vivo islet studies were drawn from separate colonies and included male and female C57BL/6 mice that were *Nnt* WT, heterozygous, or mutant, interchangeably. Mice were housed on a 12 hour light/dark cycle with ad libitum access to chow (PicoLab Rodent Diet 20 5053, LabDiet) and water and studied at 8–14 weeks of age. Both male and female mice were tested. Occasional malocclusion was present in this colony; nondiabetic mice with adult body weight of less than 20 grams were excluded from analysis. High fat diet pellets fed to female mice were 60% lard, obtained from Harlan/Envigo (TD.06414).

### Metabolic testing.

Nonfasting blood glucose (ReliOn meter) and plasma insulin (Crystal Chem) were measured from tail-tip blood samples. Intraperitoneal Glucose tolerance tests (GTTs) and insulin tolerance tests (ITTs) were performed as previously described (3). Mice were fasted for 5 hours before GTT (2 g/kg; Hospira), while ITT (1.5 U/kg; Humulin-R, Eli Lilly) was performed in the fed state. Body composition (fat/lean mass) was measured by the University of Massachusetts Medical School Mouse Metabolic Phenotyping Center (UMMS MMPC) using proton magnetic resonance spectroscopy (1H-MRS) (Echo Medical System). Metabolic testing was performed on both male and female mice. Since female *Irs2^–/–^* mice remained nondiabetic, subsequent in vivo histological and mechanistic experiments to investigate the rescue by *Cdk4-R24C* could only be performed on male mice.

### Hyperinsulinemic-euglycemic clamps.

Clamp studies were performed in the UMMS MMPC. Briefly, jugular vein catheters were implanted and mice were allowed to fully recover. Mice were fasted overnight, then primed with an initial insulin bolus (150 mU/kg) followed by a continuous 2 hour infusion at a rate of 15 pmol/kg/min. 20% glucose was infused at variable rates to maintain basal glucose levels, and blood glucose samples were taken at 10–20 minute intervals.

### Pancreas histology.

Mice were injected with BrdU (50 g/kg i.p.) at 4 hours and 2 hours before euthanasia. Pancreata were dissected, fixed in 10% formalin (Sigma-Aldrich) for 4 hours, embedded in paraffin, and cut in 5 μm sections. Sections were stained as described (77); the antibodies used (insulin, 1:200; glucagon, 1:100; BrdU, 1:200; pHH3, 1:100; PDX1, 1:100; ALDH1A3, 1:100; FOXO1, 1:100) and unmasking conditions are detailed in Supplemental Table 3. β cell mass was quantified using full-pancreas sections histochemically stained for insulin (DAB) and hematoxylin and scanned. Mass was calculated as the product of the wet weight of the pancreas and the percentage insulin-labeled area quantified using Adobe Photoshop and Image J (17). β cell proliferation was expressed as the percentage insulin-labeled cells that colabeled for BrdU or pHH3 (17) using cell profiler pipelines developed and implemented by a blinded, unbiased lab member (98). TUNEL staining was performed using the Promega DeadEnd Fluorometric system as previously described (1).

### Bulk RNA-Seq.

Islets were isolated from male mice by ductal collagenase (Roche) injection and Ficoll (Histopaque-1077; Sigma-Aldrich) gradient as previously described (1). RNA was isolated using the Norgen all-in-1 kit and sent to Quick Biology for library preparation and sequencing on the Illumina HiSeq 4000 with paired-end 150 bp reads. Sequence data are deposited in the GEO repository under accession number GSE235129. Paired-end reads were aligned to the mouse genome mm10 using the STAR aligner (99). Raw gene counts for each sample were generated from the subsequent bam files using HTSeq (100). Downstream analysis including filtering, normalization, and differential expression of the RNA-Seq counts was performed in edgeR (101). Principal component analysis and K-means clustering (2,000 most variable genes, 10 clusters) were performed using the iDEP platform (102).

### Islet Isolation and Culture.

Islets were isolated as above from adult (10–40 week) C57BL/6J or C57BL/6N (*Nnt* genotypes NN, NJ, or JJ were used interchangeably) male and female mice and plated overnight in islet complete media (ICM: RPMI, 10% FBS [Sigma-Aldrich], penicillin/streptomycin, 5mM glucose). The following day, islets were handpicked, trypsinized to single cells (0.05% trypsin), and plated on glass coverslips for immunofluorescence or plastic 24 well plates for protein/RNA analyses. A day after trypsinization, islet cells were exposed to adenovirus and/or drug for 72 hours in 15 mM glucose unless otherwise specified. Adenoviruses were purchased from Vector Biolabs, and a list of all adenoviruses and MOI used is detailed in Supplemental Table 4. The m-Foxo1-ADA and h-CDK4-R24C adenoviruses were created from plasmid (Addgene no. 12143 and no. 11254) (103). Chemicals used include Leptomycin B (100 nM; Sigma-Aldrich), MK-2206 (5 μM; Selleckchem), C646 (25 μM; Selleckchem), MB-3 (10 μM; Santa Cruz Biotechnology), salermide (25 μM; Tocris), vorinostat/SAHA (5 μM; Selleckchem), and sodium butyrate (0.5mM; Selleckchem). For starvation experiments, islet cultures were cultured in RPMI-based media prepared exactly as for ICM, except containing 2 mM glucose without FBS for 16 hours before harvesting. After the experiments, cells were fixed for immunofluorescence as described below, harvested for RNA and protein with SKP buffer with β-mercaptoethanol per protocol (Norgen), or for protein with lysis buffer with PhosSTOP (Sigma-Aldrich) as described (15).

### Glucose stimulated insulin secretion.

100 IEQ of dispersed mouse islet cells were plated in 24 well plates and allowed to recover overnight. The following day, cells were transduced with the indicated adenoviruses and incubated in 15 mM glucose for 72 hours. Glucose-stimulated insulin secretion assays were performed as described before (3) with some modifications. Briefly, cells were washed twice and preincubated in Krebs buffer (10 mM HEPES, 1.19 mM MgSO_4_, 119 mM NaCl, 4.74 mM KCl, 1.19 mM KH_2_PO_4_, 2.54 mM CaCl_2_-2H_2_O, and 25 mM NaHCO_3_, pH 7.4, 95% O_2_) with 3% BSA and 2.8 mM glucose for 60 minutes at 37°C. Cells were then incubated in Krebs buffer with 1% BSA and 3 mM glucose, followed by Krebs buffer with 1% BSA and 20 mM glucose, each for 30 minutes at 37°C. After each glucose incubation, the buffer was removed and stored at –20°C for insulin analysis. Finally, cells were lysed in extraction buffer (0.18 M HCl in 70% ethanol), scraped from the wells, sonicated for 2 pulses (30/30 seconds, Ampl 30%) in a Q800R sonicator (Qsonica), and stored at –20°C for total insulin content analysis.

### Quantitative PCR.

RNA was isolated from dispersed islet experiments using the Norgen All-in-1 kit. cDNA synthesized with SuperScript IV VILO (Thermo Fisher Scientific) was amplified with PerfeCTa SYBR (VWR) using either Eppendorf or BioRad thermocyclers (15) and analyzed by the ΔΔCT method. Primers are in Supplemental Table 5.

### Western blotting.

Immunoblots were performed as described (15). Briefly, dispersed islet cells were harvested either with the Norgen All-in-1 kit or with Lysis Buffer (0.5M Tris pH 6.8, 10% SDS, 100mM DDT, 10mg/ml APMSF, and protease inhibitor), both supplemented with PhosSTOP (Sigma-Aldrich). Protein lysates were separated by SDS-PAGE, transferred to PVDF membrane (BioRad), and blocked in either 5% milk or BSA (w/v) in PBS with 0.1% Tween-20. Primary antibodies used are described in Supplemental Table 6. Data was collected on film or using a BioRAD gel doc station, using ECL (Thermo Fisher Scientific), ECL Prime (GE Healthcare), or SuperSignal West Femto (Thermo Fisher Scientific), and bands were quantified using ImageJ. See complete unedited blots in the supplemental material.

### Cultured islet cell immunofluorescence.

Dispersed mouse islets were fixed in 4% PFA for 10 minutes and stored in PBS at 4°C. Fixed cells on coverslips were immunostained as described (15). Briefly, coverslips were blocked in goat serum–based block plus 0.1% Triton-X, then stained with the following antibodies: Insulin (1:200; Dako) and FOXO1 (1:50; Cell Signaling Technology), followed by Alexa Fluor secondary antisera (Invitrogen), and coverslips were mounted onto slides using FluoroshieldÔ with Dapi (Sigma-Aldrich). Coverslips were imaged on either a Leica SP-5 Laser Scanning Confocal Fluorescence microscope, TissueGnostics Fluorescence Slide Scanner, or a Nikon TE2000-E2 inverted microscope. Quantification of nuclear FOXO1 was performed by a blinded individual utilizing a trinary scoring system.

### Statistics.

Data were analyzed using GraphPad Prism. Data are represented as mean SEM unless specified otherwise. *P* values were calculated using 2-tailed *t* test, 1-way ANOVA, or 2-way ANOVA, as specified. *P* < 0.05 was considered significant.

### Study approval.

All mouse studies were approved by the Institutional Animal Care and Use Committees of the University of Pittsburgh, University of Massachusetts Medical School, and Weill Cornell Medicine.

### Data availability.

The RNA-Seq data are available in the GEO database under accession number GSE235129. All other data are available in the Supporting data values XLS file.

## Author contributions

RES performed the majority of the experiments, contributed to study design and conceptualization, data analysis, data interpretation, funding acquisition, and manuscript preparation. HVLG performed Western blots, qPCR, and insulin secretion assays. RBS contributed to the in vivo metabolic studies and assisted with the RNA-seq studies. CD performed some histology and microscopy experiments. DR performed the initial RNA-seq data analyses. SGR provided the *CDK4-R24C* mice as well as intellectual contributions to data interpretation. LCA conceptualized the study and contributed to data analysis and interpretation, funding acquisition, and manuscript preparation. All authors had the opportunity to review and edit the manuscript.

## Supplementary Material

Supplemental data

Supplemental table 1

Supplemental table 2

Supporting data values

## Version 1 09/15/2023

Electronic publication

## Version 2 12/20/2023

fixed amount of NaCl in Krebs buffer in Methods
