# Supplementary material for: Noncanonical CDK4 signaling rescues diabetes in a mouse model by promoting β cell differentiation
Source: J Clin Invest. 2023 Sep 15;133(18):e166490. doi: 10.1172/JCI166490 (PMC10503800; doi:10.1172/JCI166490)
Supplement: Supplemental data [file jci-133-166490-s009.pdf]

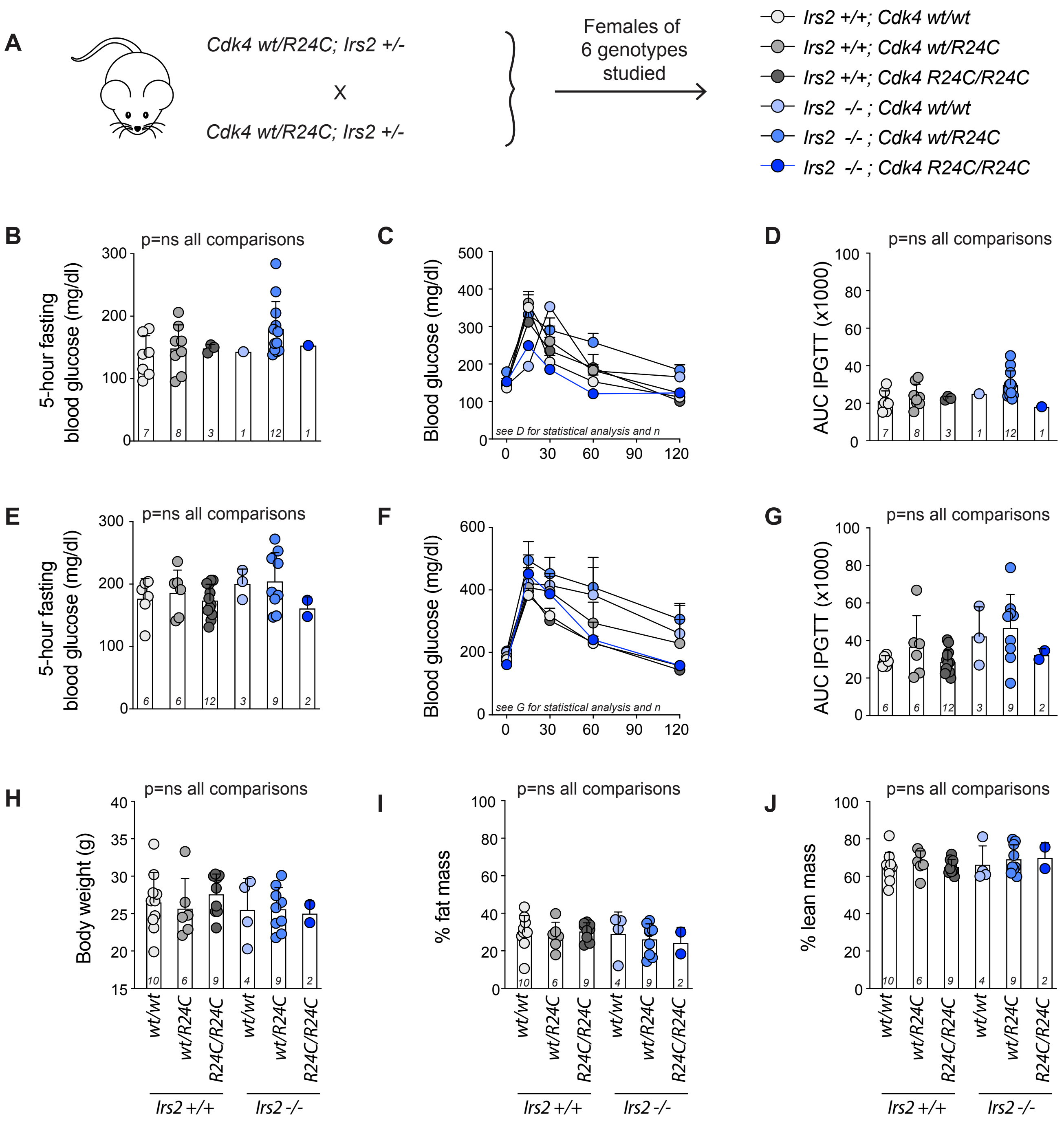

Supplemental Figure 1. Metabolic assessment of *Irs2*-/- female mice without or with replacement of *Cdk4* with *Cdk4-R24C*. A: Breeding strategy and experimental genotypes. Panels B-D show data obtained from 14-week-old females fed regular chow: 5-hour-fasting blood glucose (B) and blood glucose time course (C) or AUC (D) after intraperitoneal glucose challenge. Number of replicates is shown for each genotype. Since *Irs2*-/- females were not diabetic in normal chow conditions we stopped this experiment and repeated the study after 4 weeks of high fat feeding (E-J). Number of replicates is shown for each genotype. 5-hour fasting blood glucose did not identify hyperglycemia in *Irs2*-/- females on HFD (E). Glucose challenge identified mild hyperglycemia in *Irs2-/-; Cdk4-wt/R24C* females that was not statistically different from controls by ANOVA of AUC (G), so the experiment was stopped for futility to test for rescue by *R24C/R24C* since diabetes did not manifest in *Irs2*-/- animals. Body composition analysis of females after 4 weeks HFD by 1H-MRS Echo-MRI found no difference in body weight (H), % lean mass (I) or % fat mass (J). Statistics are by one-way ANOVA with Tukey post-test. *p<0.05; **p<0.01; ***p<0.001; ****p<0.0001.

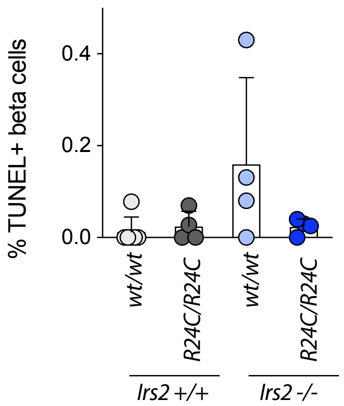

Supplemental Figure 2. TUNEL staining showed a non-significant reduction in apoptosis in *Cdk4-R24C/R24C* beta cells. Pancreas sections from male mice were labeled by TUNEL and immunostained for insulin and dapi, then blinded images quantified for the % of insulin+ cells that co-labeled for TUNEL (n ≥ 4). All comparisons nonsignificant with p>0.05.

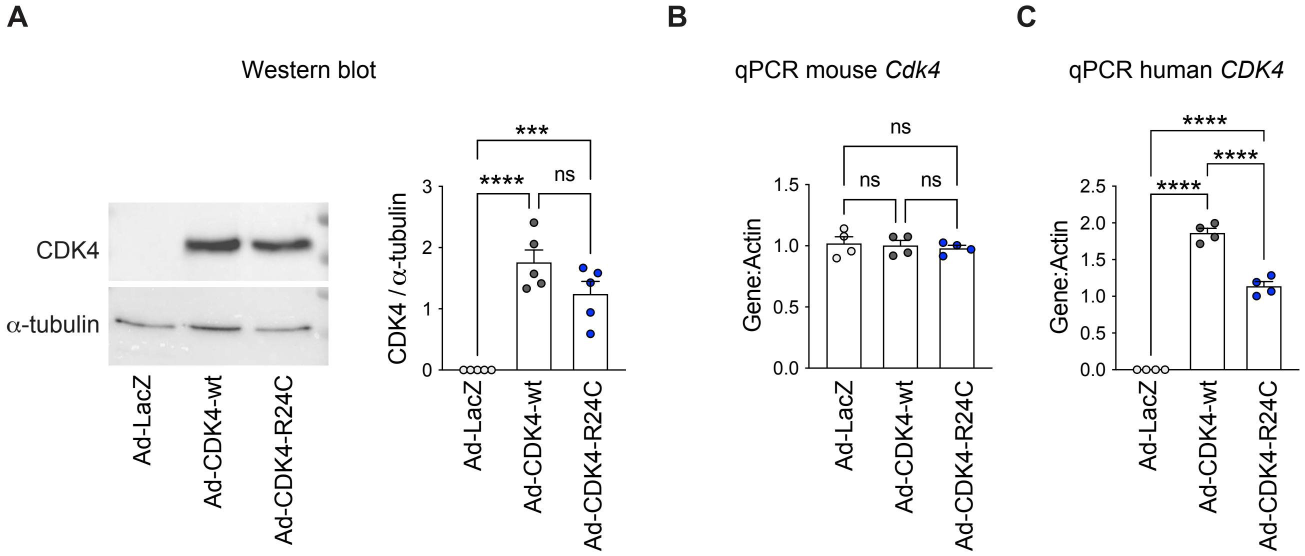

Supplemental Figure 3. Overexpression of WT and R24C variants of human CDK4 in mouse islet cells results in similar protein abundance. A-C: Dispersed mouse islet cells were transduced with the indicated adenoviruses, all with a multiplicity of infection (MOI) of 5, and cultured in 15 mM glucose for 72 hours, then lysed and processed for immunoblot (A) (n=5) or qPCR (B-C) (n=4). Statistics are by one-way ANOVA with Tukey post-test. ***p<0.001; ****p<0.0001.

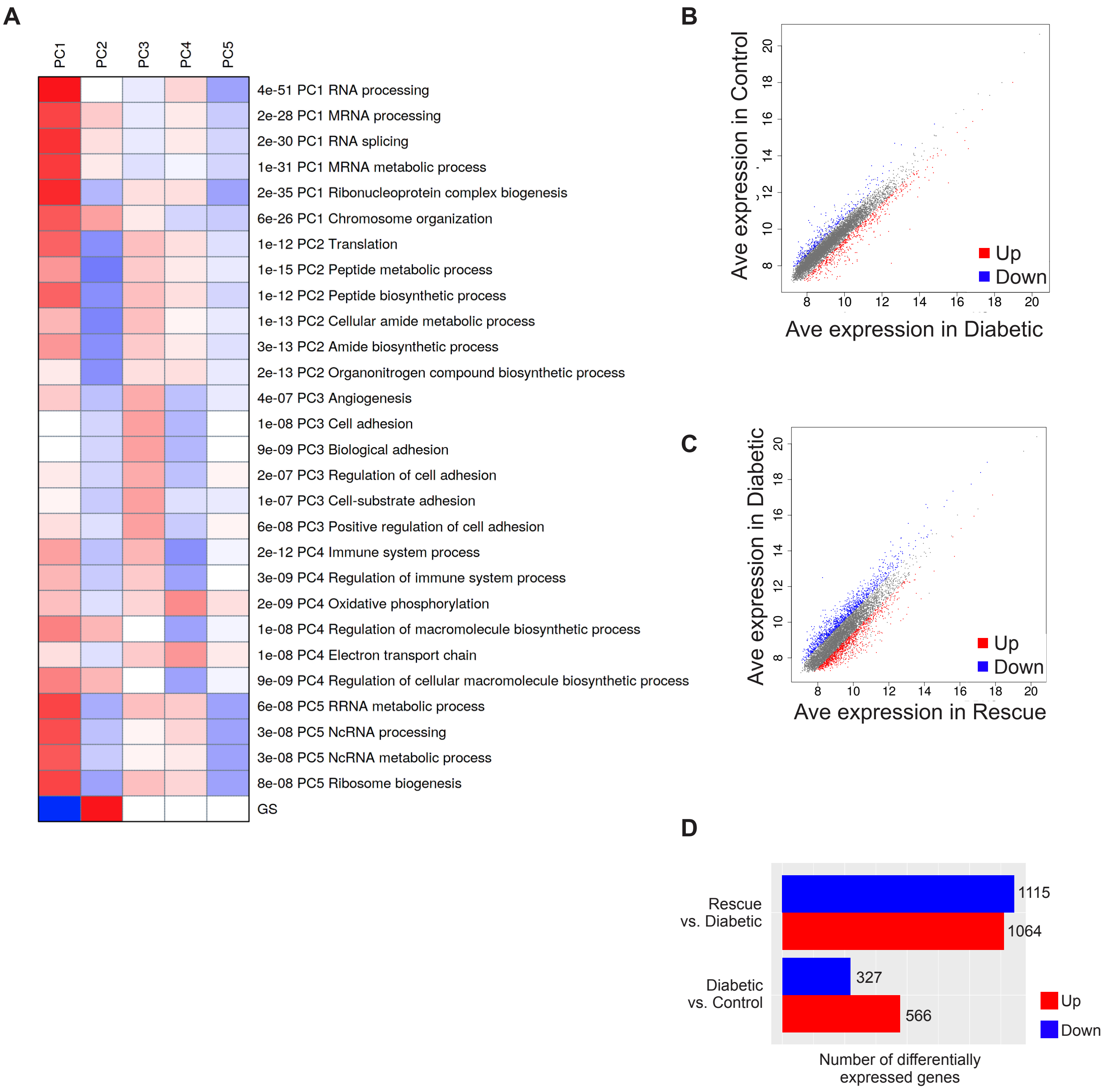

Supplemental Figure 4. RNA sequencing of whole islets directly after isolation revealed numerous gene expression changes between groups. A: Goes with the principal component analysis shown in Figure 3C. Intriguingly, PC1 is dominated by RNA processing related genes, and PC2 contains genes related to peptide biosynthesis. B-D: Scatterplots comparing Irs2+/+; *Cdk4-wt/wt* (Control) versus *Irs2-/-;Cdk4-wt/wt* (Diabetic) (B) or *Irs2-/-;Cdk4-wt/wt* (Diabetic) versus *Irs2-/-;Cdk4-R24C/R24C* (Rescue) (C) show numerous gene changes both upregulated and downregulated; changes are quantified in (D).

A

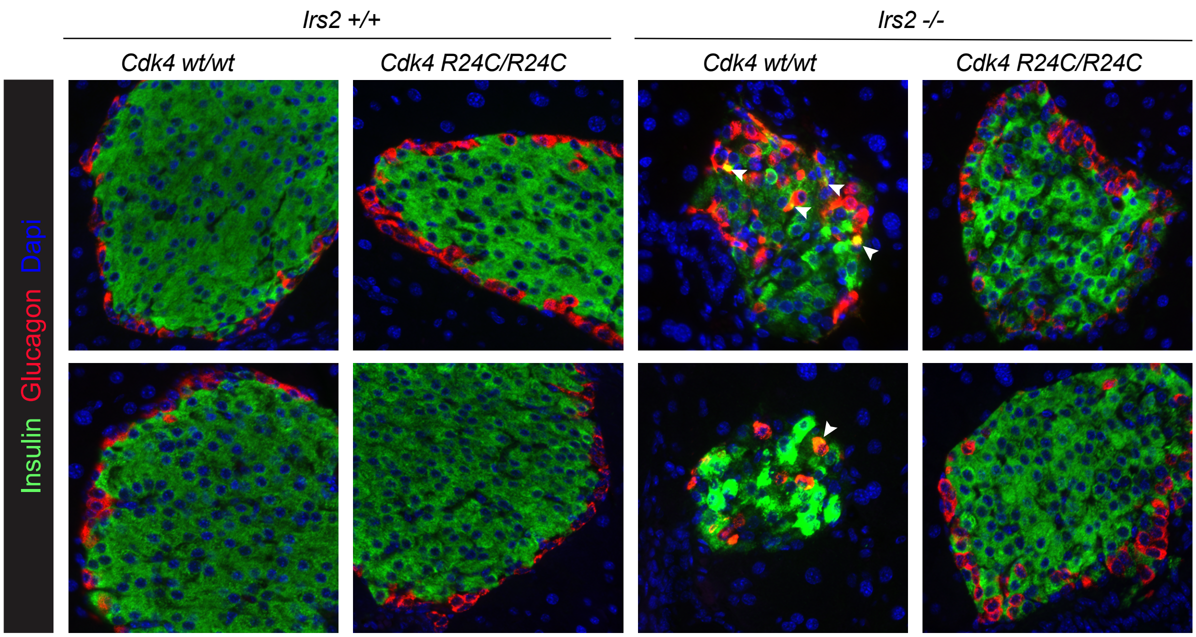

B

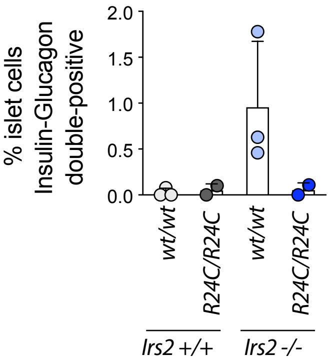

Supplemental Figure 5. Insulin-Glucagon double-positive cells observed in *Irs2*-/- islets were rescued by *CDK4-R24C*. A: Pancreas sections from the indicated genotypes were immunostained for insulin, glucagon and dapi. Original magnification 200X. Representative images from two different animals from each genotype are shown. Some images in (A) are repeated from Figure 3A. B: Quantification revealed an increased number of insulin-glucagon double-staining cells in Irs2-/- sections, which was restored to normal in *Irs2*-/- mice with two alleles of *Cdk4 R24C*, n=2-3.

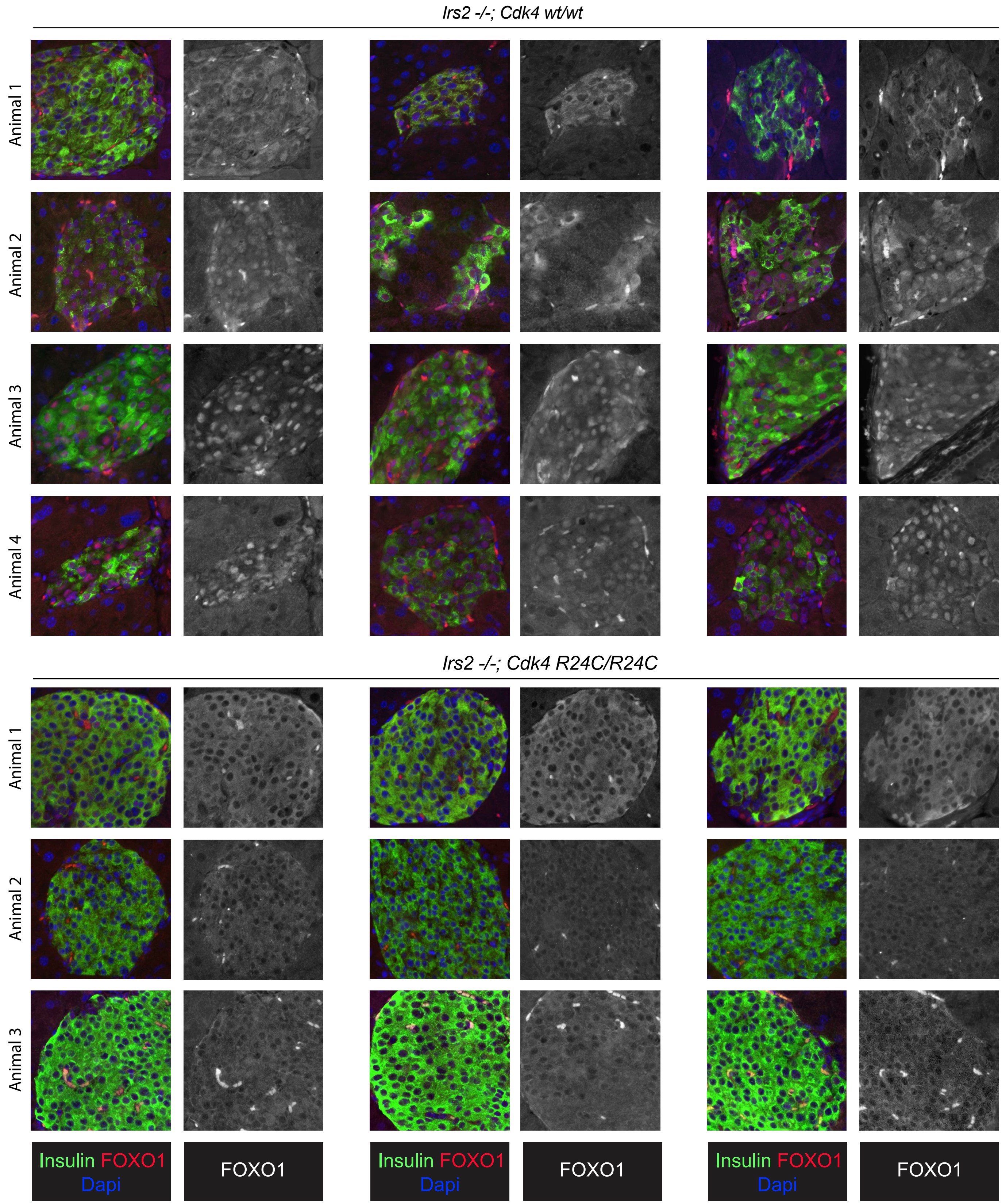

Supplemental Figure 6. FOXO1 localization is often nuclear in Irs2-/- beta cells, but mostly excluded from nuclei in Irs2-/-; CDK4-R24C/R24C beta cells. Pancreas sections from the indicated genotypes were immunostained for FOXO1 (red), insulin (green),and dapi (blue). Adjacent to each RGB image is a greyscale of FOXO1 alone. Original magnification 200X. Three representative islets from each animal are shown.

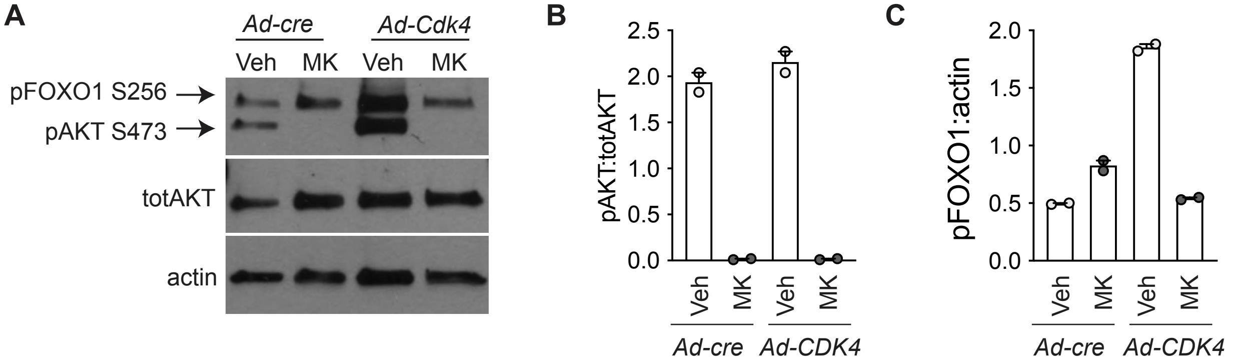

Supplemental Figure 7. CDK4 overexpression increases FOXO1 phosphorylation through an indirect mechanism that requires AKT. Mouse islet cells cultured in 15mM glucose were transduced with Ad-cre (control virus) or Ad-CDK4 for 48 hours, followed by 24h exposure to the MK-2206 AKT inhibitor or vehicle. (A) Lysates were subjected to immunoblotting with antisera against phosphorylated FOXO1 (S256), phosphorylated AKT (S473), total AKT, or Actin. Quantification showed that CDK4 overexpression did not alter the ratio of p-AKT to total AKT, and that MK-2206 reduced p-AKT regardless of CDK4 overexpression (B) (n=2). On the other hand, CDK4 overexpression markedly increased p-FOXO1 (C), but inhibition of AKT with MK-2206 completely prevented the CDK4-induced increase in p-FOXO1 (n=2).

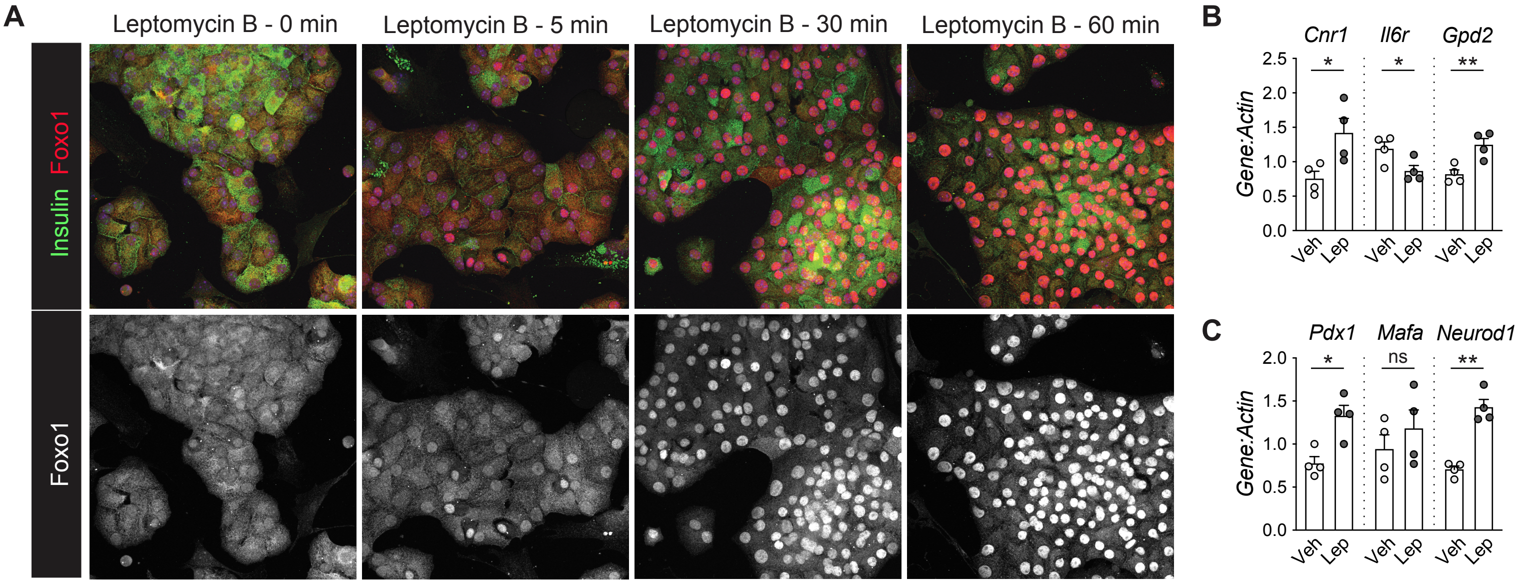

Supplemental Figure 8. Forced nuclear accumulation of FOXO1 is not sufficient to repress Pdx1. A: Dispersed mouse islet cells cultured in ICM with 15mM glucose were treated with nuclear export inhibitor Leptomycin B (100nM) for the indicated duration, then fixed, immunostained for insulin (green), FOXO1 (red) and DAPI (blue), and imaged by confocal microscopy. The red (FOXO1) channel is displayed separately below. Original magnification 400X. B-C: Dispersed mouse islet cells treated with Leptomycin B for 24 hours were analyzed by qPCR for FOXO1 targets (B) or beta cell maturation genes (C). (A) contains representative images; number of replicates is shown for all other panels. Statistics (B, C) are by unpaired T-test. *p<0.05, **p<0.01.

For **Supplementary Tables 1** and **2** please see separate .xlsx files

Supplementary Table 3. Antibodies used for immunofluorescence

Antibody	Dilution	Vendor	Cat No.	Unmasking
ALDH1A3	1:100	Novus	NBP1-91657	Citrate
BrdU	1:200	Abcam	ab6326	HCl
FOXO1	1:100	Cell Signaling Technology	2880	Tris-EDTA
glucagon	1:100	Cell Signaling Technology	2760	N/A
insulin	1:200	Dako	A0564	N/A
PDX1	1:100	Abcam	ab47267	N/A
pHH3	1:100	Cell Signaling Technology	3377	Tris-EDTA

Supplementary Table 4. Adenoviruses used in the study, with multiplicity of infection (MOI). Final MOI was kept constant across all experiments; where viruses were combined, the MOI difference was made up with control virus. Mammalian species of origin are labeled as mouse (m) or human (h).

Virus	MOI
Control (Ad-Cre)	5-20
Control (Ad-LacZ)	5-20
Ad-h-CDK4	5
Ad-h-CDK4-R24C	5
Ad-m-Cyclin D2	5
Ad-m-Foxo1	10
Ad-m-Foxo1-ADA	10
Ad-m-p16	5
Ad-m-shE2f1	10

Supplementary Table 5. qPCR primers

Gene	Forward	Reverse
Cdk4-mouse	ATGGCTGCCACTCGATATGAA	TCCTCCATTAGGAACTCTCACAC
Cdk4-human	CTGGTGTTTGAGCATGTAGACC	GATCCTTGATCGTTTCGGCTG
Cnr1	AAGTCGATCTTAGACGGCCTT	TCCTAATTTGGATGCCATGTCTC
E2f1	GCCCTTGACTATCACTTTGGTCTC	CCTTCCCATTTTGGTCTGCTC
Foxo1	TGCTGTGAAGGGACAGATTG	GAGTGGATGGTGAAGAGCGT
Gpd2	GAAGGGGACTATTCTTGTGGGT	GGATGTCAAATTCGGGTGTGT
Il6r	CCTGAGACTCAAGCAGAAATGG	AGAAGGAAGGTCGGCTTCAGT
Ins1	ACCTTTGTGGTCCTCACCTG	AGCTCCAGTTGTGGCACTTG
Ins2	TGTGGTTCTCACTTGGTGGA	CTCCAGTTGTGCCACTTGTG
Ki67	CTGCCTGCGAAGAGAGCATC	AGCTCCACTTCGCCTTTTGG
Mafa	GAGGAGGTCATCCGACTGAAA	GCACTTCTCGCTCTCCAGAAT
Neurod1	GCAGCTCTGGAGCCCTTCTT	GCGGCACCGGAAGAGAAGAT
Ngn3	CTGCGCATAGCGGACCACAGCTTC	CTTCACAAGAAGTCTGAGAACACCAG
Nkx6.1	CTTCTGGCCCGGAGTGATG	GGGTCTGGTGTGTTTTCTCTTC
Pcna	ACCTGCAGAGCATGGACTCG	GCAGCGGTATGTGTCGAAGC
Pdx1	GATGAAATCCACCAAAGCTCA	GAATTCCTTCTCCAGCTCCA

Supplementary Table 6. Antibodies used for immunoblotting

Antibody	Dilution	Vendor	Cat No.
actin	1:2000	Sigma	MAB1501
α-tubulin	1:2000	Calbiochem	#CP06
CDK4	1:1000	Proteintech	#11026-I-AP
FOXO1	1:500	Cell Signaling Technology	#2280
pFOXO1 S256	1:500	Cell Signaling Technology	#84192
pSIRT1	1:500	Novus	JJ206-6

Full unedited gel for Figure 4H

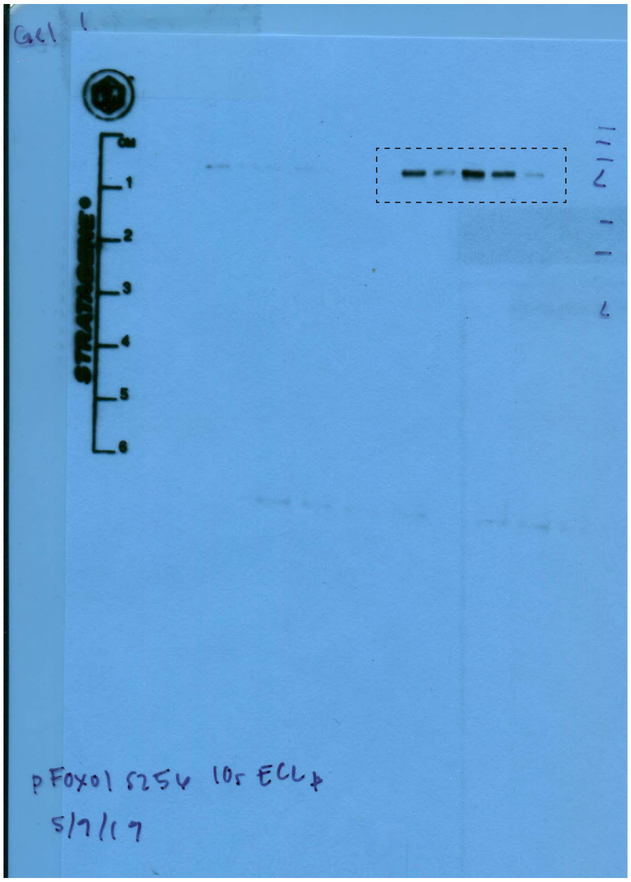

pFOXO1 S256

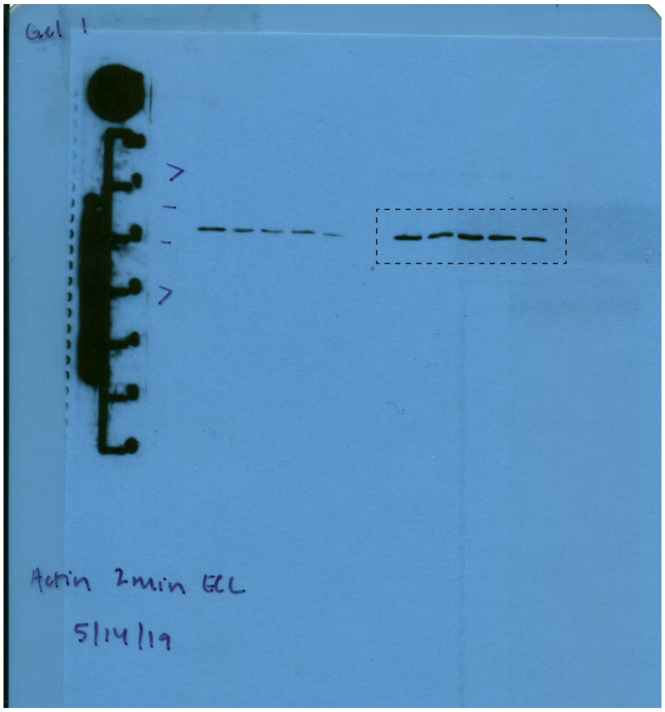

Actin

Full unedited gel for Figure 7G

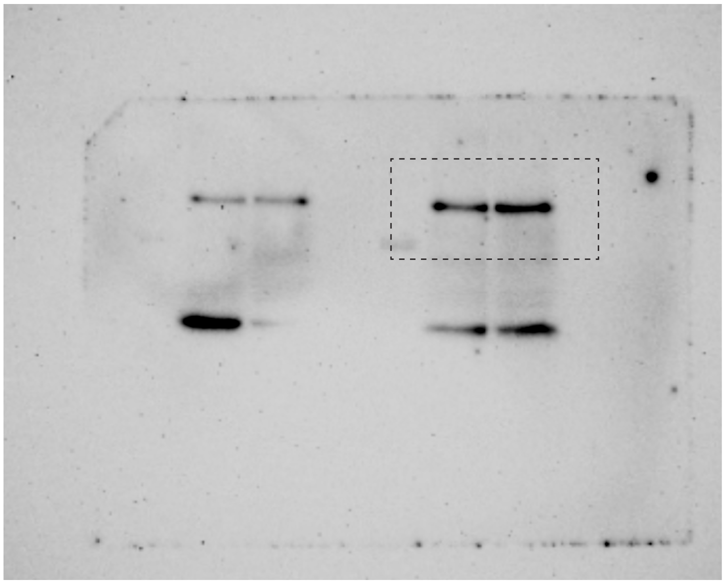

p-SIRT1

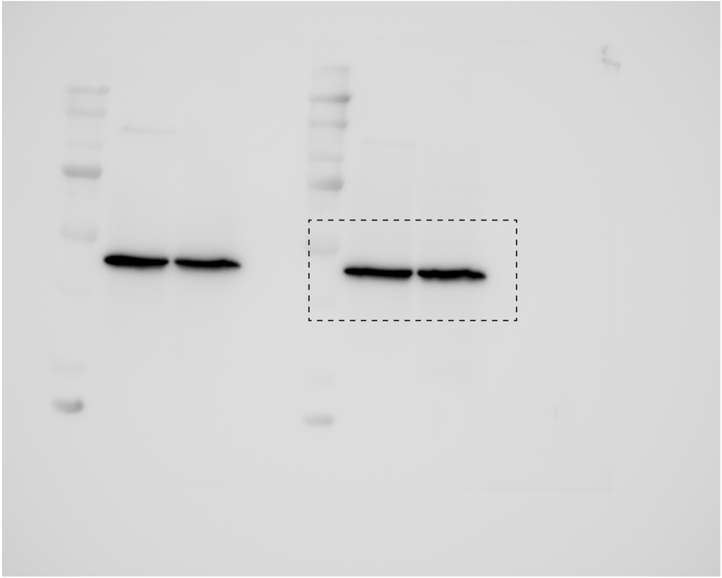

Actin

Full unedited gel for Figure 7H

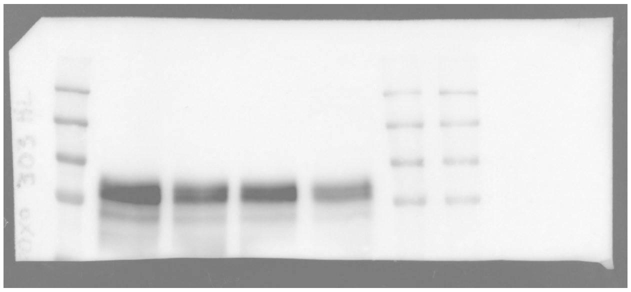

FOXO1- composite

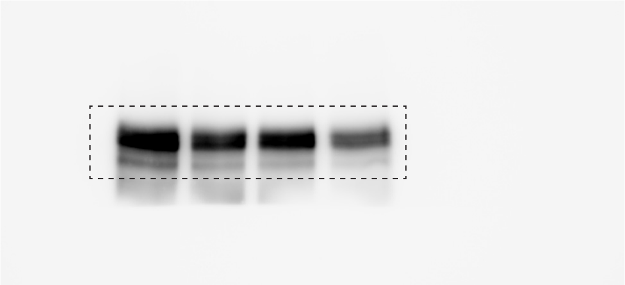

FOXO1- chemiluminescence

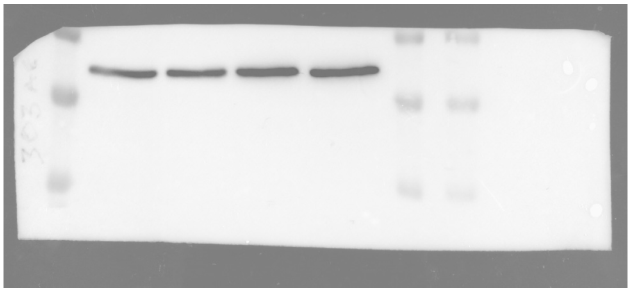

Actin- composite

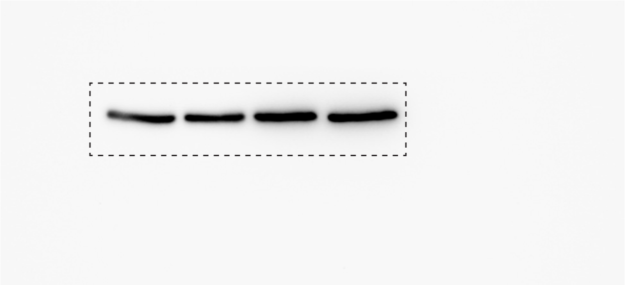

Actin- chemiluminescence

Full unedited gel for Supplemental Figure 3A

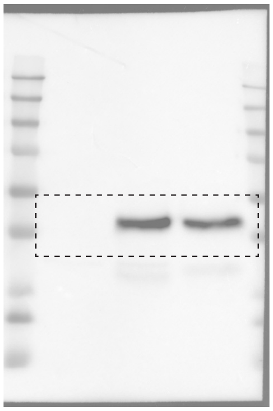

CDK4
composite

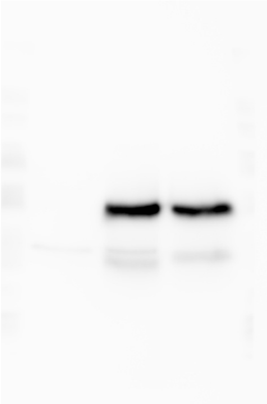

CDK4
chemiluminescent

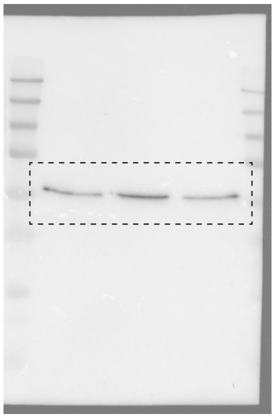

alpha tubulin
composite

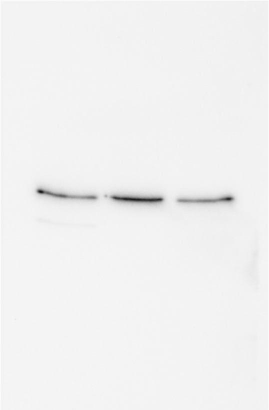

alpha tubulin
chemiluminescent

Full unedited gel for Supplemental Figure 7A

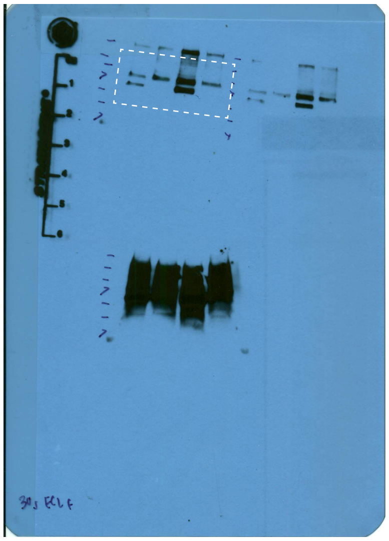

pFOXO1 S256
pAKT S473

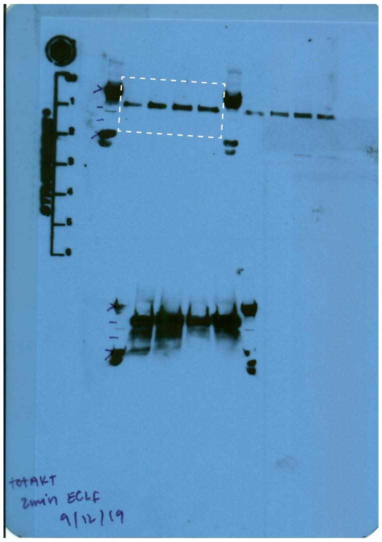

total AKT

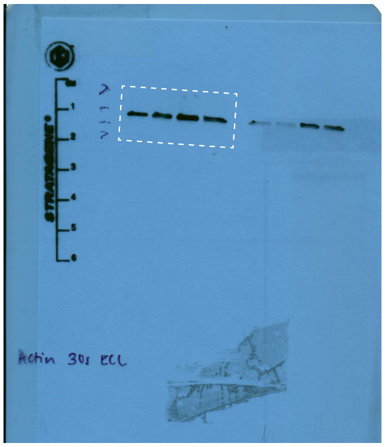

Actin
